# Periodontal Status and Herpesiviridae, Bacteria, and Fungi in Gingivitis and Periodontitis of Systemically Compromised Pediatric Subjects: A Systematic Review

**DOI:** 10.3390/children12030375

**Published:** 2025-03-17

**Authors:** Federica Di Spirito, Massimo Pisano, Maria Pia Di Palo, Giuseppina De Benedetto, Iman Rizki, Gianluigi Franci, Massimo Amato

**Affiliations:** Department of Medicine, Surgery and Dentistry, University of Salerno, Via S. Allende, 84081 Baronissi, Italy; pisano.studio@virgilio.it (M.P.); mariapia140497@gmail.com (M.P.D.P.); giusydb15@gmail.com (G.D.B.); i.rizki@studenti.unisa.it (I.R.); mamato@unisa.it (M.A.)

**Keywords:** herpesviridae, gingivitis, periodontitis, aggressive periodontitis, mouth, bacteria, virus, fungi, child

## Abstract

Background: Gingivitis and periodontitis are microbially associated diseases, with some features characteristic of pediatric age and others linked to systemic diseases. While the role of periodontal pathogenic bacteria is well recognized, the contribution of fungi and viruses, particularly *Herpesviridae*, remains controversial. Studies in adults have highlighted the presence of *Herpesviridae*, but evidence in pediatric subjects, especially systemically compromised, is limited. This systematic review aimed to assess periodontal status (e.g., health, gingivitis, periodontitis, necrotizing gingivitis, and/or periodontitis) and the subgingival and/or salivary microbial (bacterial, viral, and fungal) profile in systemically compromised pediatric (≤18 years) subjects with gingivitis and/or periodontitis compared to clinical periodontal health. Methods: The review protocol was registered on PROSPERO (CRD42024597695) and followed the PRISMA statement. Data from eight studies were descriptively analyzed and qualitatively assessed through ROBINS-I and JBI tools. Results: CMV was frequently detected, particularly in necrotizing gingivitis (19.40%). EBV was found in necrotizing gingivitis (20.69%) and periodontitis (10.34%); HSV was mainly associated with gingivitis and necrotizing gingivitis. Bacteria species in periodontitis included *Porphyromonas gingivalis*, *Tannerella forsythia*, *Fusobacterium*, and *Campylobacter* species. *Candida albicans* was detected in periodontitis, suggesting a fungal involvement in the disease’s pathogenesis. Although the bacterial and fungal profile was not investigated, limited viral presence was noted in subjects with healthy periodontium, indicating a stable microbiome. Conclusions: These findings underscore the dynamics of microbial interactions in the progression of periodontal disease in systemically compromised pediatric subjects.

## 1. Introduction

Periodontitis and gingivitis are inflammatory diseases affecting the supporting structures of the teeth [1,2]. According to the 2017 classification of Periodontal and Peri-Implant Diseases and Conditions, co-presented by the American Academy of Periodontology (AAP) and the European Federation of Periodontology (EFP), gingivitis refers to a reversible inflammation of the gingiva caused by plaque accumulation, characterized by redness, swelling, and bleeding without clinical attachment or alveolar bone loss [1,3,4]. In contrast, periodontitis involves the irreversible destruction of the periodontal ligament and alveolar bone, leading to clinical attachment loss, pocket formation, and potential tooth loss [1,3,4].

The AAP and EFP classification defined necrotizing gingivitis as a severe inflammatory condition characterized by rapid onset and destruction of the gingival tissues, presenting as necrosis of the interdental papillae, spontaneous bleeding, and pain [1,5,6]. It is associated with specific risk factors, including immunosuppression, malnutrition, and poor oral hygiene, and is frequently observed in vulnerable populations [1,5,6]. The condition is commonly linked to an overgrowth of specific anaerobic bacteria, including *Fusobacterium* and *Prevotella species*, along with host immune dysfunction [1,5,6].

In the pediatric population, gingivitis is commonly associated with plaque accumulation and is reversible with proper oral hygiene [7,8]. In contrast, periodontitis, although less frequent, assumes clinical significance, especially in children with systemic, genetic, or immunological conditions, where it often presents as an aggressive and destructive condition [8,9].

Periodontal diseases result from a complex interplay between microbial dysbiotic biofilms and the host immune inflammatory response, influenced by genetic and environmental factors [10,11,12].

In a healthy oral environment, the subgingival microbiota maintains a balanced composition dominated by early colonizers, like *Streptococcus species* and *Actinomyces*, by contributing to stable biofilm formation [13,14]. However, in gingivitis and periodontitis, the microbial composition shifts toward dysbiosis, characterized by an increase in pathogenic bacteria belonging to the red and orange complexes, such as *Porphyromonas gingivalis*, *Tannerella forsythia*, *Treponema denticola*, and *Fusobacterium nucleatum* [14,15,16]. These pathogens play key roles in the pathogenesis of periodontitis by producing proteolytic enzymes, modulating host immune responses, and releasing lipopolysaccharides that exacerbate inflammation and tissue destruction [11,14,17]. Additionally, *Aggregatibacter actinomycetemcomitans* has been strongly associated with periodontitis in pediatric populations due to its virulence factors, such as leukotoxins that induce neutrophil apoptosis and promote inflammation [18,19,20].

Beyond the role of bacteria, emerging evidence has highlighted the potential role that certain viruses, particularly members of the *Herpesviridae* family, may play in the pathogenesis of periodontitis and gingivitis [21,22].

These viruses can remain latent in epithelial cells and leukocytes, reactivating under conditions of stress, immunosuppression, or microbial dysbiosis [12,21,23]. Reactivation amplifies the inflammatory response and contributes to tissue destruction [12,21,23]. Evidence suggests that CMV and EBV are frequently detected in periodontally affected sites, where their interactions with bacterial pathogens create a synergistic environment that accelerates disease progression [12,21,23].

Fungi, though less investigated, may also contribute to the complexity of the oral microbiome. *Candida albicans* has been identified in periodontal lesions, where it may exacerbate inflammation through the production of toxic metabolites and synergistic interactions with bacterial pathogens [24,25].

The 2017 World Workshop highlighted that several systemic conditions significantly impact the periodontium through immune, metabolic, and structural mechanisms [3,4,26].

Systemic diseases often impair host responses, increasing susceptibility to tissue destruction and attachment loss, which may result in periodontal disease and conditions [26].

For instance, endocrine and metabolic disorders, such as uncontrolled diabetes, worsen systemic and local inflammation, while chronic periodontal inflammation impairs glycemic control, creating a bidirectional relationship between diabetes and periodontitis [26,27]. Immunodeficiencies, both congenital and acquired, further increase periodontal vulnerability and compromise the host’s ability to control pathogenic biofilms, leading to rapid disease progression, even in cases of moderate biofilm accumulation [26,28]. Genetic conditions such as Down syndrome are associated with a higher prevalence of early-onset periodontitis due to neutrophil dysfunction and abnormal T-cell responses [26,29]. Similarly, rare conditions like Papillon–Lefèvre syndrome disrupt antimicrobial neutrophil functions, exacerbating inflammation and tissue destruction [26].

Overall, systemic diseases not only heighten susceptibility to periodontitis but also alter the composition and activity of the oral microbiome [30,31]. These changes create an environment that may be conducive to synergistic interactions among bacteria, viruses, and fungi.

Despite increasing awareness of the relationship between periodontitis and systemic conditions, the interplay among bacteria, viruses, and fungi in pediatric patients remains underexplored [30,31]. This gap might be particularly relevant for children with systemic diseases, where immunosuppression, inflammatory status, genetic predisposition, or metabolic dysfunction may amplify the complexity of periodontal microbial biofilm, alter inter-species interactions, and accelerate the progression of periodontal disease.

The null hypothesis states that there are no relevant differences in salivary and/or subgingival microbial (bacterial, viral, and fungal) profiles between systemically compromised pediatric subjects with gingivitis and/or periodontitis and those with clinically healthy periodontium. The alternative hypothesis, conversely, postulates that relevant differences in salivary and/or subgingival microbial (bacterial, viral, and fungal) profiles exist between systemically compromised pediatric subjects with gingivitis and/or periodontitis and those with clinically healthy periodontium.

Therefore, the present systematic review aimed to evaluate periodontal status (e.g., health, gingivitis, periodontitis, necrotizing gingivitis, and/or periodontitis) and the subgingival and/or salivary microbial (bacterial, viral, and fungal) profile in systemically compromised pediatric subjects with gingivitis and/or periodontitis compared to clinical periodontal health.

## 2. Materials and Methods

### 2.1. Study Protocol

The study protocol was developed in alignment with the Preferred Reporting Items for Systematic Reviews and Meta-analyses (PRISMA) statement [32] and was registered on the International Prospective Register of Systematic Review PROSPERO register under registration number CRD42024597695. The protocol was established prior to initiating the literature search, data extraction, and analysis.

This research investigated the following question: “What are the periodontal status and the salivary and/or subgingival microbial (bacterial, viral, and fungal) profiles in systemically compromised pediatric subjects (≤18 years of age) with gingivitis and/or periodontitis compared to systemically compromised pediatric subjects with a healthy periodontium”?

**Figure 1:**
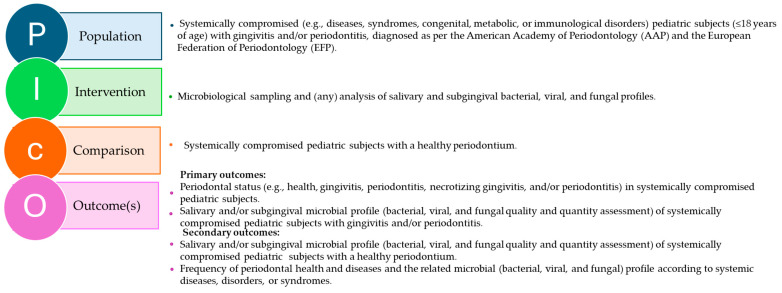
PICO model: P—population; I—intervention; C—comparison; O—outcome(s).

The question, search strategies, and inclusion/exclusion criteria were defined through the PICO model [33], as illustrated in Figure 1.

### 2.2. Search Strategy

Two reviewers (M.A. and G.F.) performed an electronic search across Scopus, MEDLINE/PubMed, Web of Science, and the Cochrane Library up to 16 July 2024. No restrictions were applied regarding publication date or status; no filters were applied. Table 1 presents the search strategy process, detailing the keywords combined with Boolean operators for each database.

### 2.3. Study Selection and Eligibility Criteria

Two independent reviewers (M.P.D.P. and M.P.) assessed the studies for eligibility, and any disagreements were resolved through discussion. A third reviewer (F.D.S.) was consulted when necessary.

The titles and abstracts from the electronic search were screened to delete duplicates or records not relevant to the topic. The full text was obtained before any possible exclusion for unclear titles and abstracts.

If the full text was unavailable, the study’s corresponding authors were contacted by e-mail.

A manual search of the reference lists of the included articles was performed to identify additional relevant records meeting the eligibility criteria.

References from the included studies were extracted and organized using Mendeley Reference Manager software, version 2.120.3, copyright Elsevier Ltd. (Amsterdam, The Netherlands).

Table 2 presents the inclusion/exclusion criteria.

### 2.4. Data Extraction and Collection

Data were independently extracted by two authors (I.R. and F.D.S.) using a standardized extraction form, which was developed prior to the completion of the literature search. For each study included in the present systematic review, the following data were extracted and collected according to the eligibility criteria:

- Study characteristics: first author, year of publication, journal, study design, quality assessment, and funding;
- Population characteristics: number of participants, mean age, gender ratio, country of origin and/or ethnicity, comorbidities, medical history of infectious diseases, and current or previous treatments for infections;
- Periodontal status: clinical, radiographic, and crevicular periodontal parameters and diagnosis;
- Microbiological (salivary and/or subgingival) sampling and analyses performed: number and type of samples collected, sampling methods, site(s), target, and microbiological identification technique;
- Outcome(s):
  - Bacterial profile: identified bacterial species from red, orange, yellow, green, and blue Sokransky complexes, along with outlier species, positivity rates, and count from PCR and/or culture analysis, and total counts of anaerobic and/or total bacteria;
  - Viral profile: HHV species detected, other viruses detected, positivity rates, and count from PCR analysis;
  - Fungal profile: type and species of fungi identified, positivity rates, and count from PCR and/or culture analysis.

### 2.5. Data Synthesis

Data from each record included were extracted and descriptively synthesized through Microsoft Excel Software 2019 (Microsoft Corporation, Redmond, WA, USA). The data synthesis process was designed as follows:

▪ To evaluate periodontal status in systemically compromised pediatric (≤18 years of age) subjects through clinical, radiographic, and crevicular periodontal parameters;
▪ To assess the prevalence of periodontal diseases in systemically compromised pediatric (≤18 years of age) subjects;
▪ To characterize the microbial (bacterial, viral, and fungal) profile in systemically compromised pediatric subjects (≤18 years of age) with gingivitis and/or periodontitis;
▪ To compare microbial (bacterial, viral, and fungal) profiles in systemically compromised pediatric subjects (≤18 years of age) with a clinically healthy periodontium;
▪ To assess the frequency of periodontal health and diseases and the related microbial (bacterial, viral, and fungal) profile according to systemic diseases, disorders, or syndromes.

### 2.6. Quality Assessment

The studies included in the present systematic review were evaluated qualitatively by two independent reviewers (G.D.B. and M.P.D.P.). The assessment employed the Risk of Bias In Nonrandomized Studies of Interventions (ROBINS-I) tool for nonrandomized studies of interventions (freely accessible online at ROBINS-I tool|Cochrane Methods) and the Joanna Briggs Institute (JBI) tools for appraising case reports and case series (freely accessible online at JBI Critical Appraisal Tools|JBI). In the case of a disagreement, a third reviewer (M.P.) was involved to assist in the resolution.

## 3. Results

A total of 297 records were retrieved from electronic databases, specifically 138 from MEDLINE/PubMed, 133 from Scopus, 23 from Web of Science, and 3 from the Cochrane Library. After removing 96 duplicates, 201 titles and abstracts were screened, and 125 records were deemed irrelevant to the study topic.

Subsequently, 76 records were selected for full-text retrieval. One record could not be retrieved despite attempts to contact the authors, and it was excluded due to a lack of response. The remaining 75 records were assessed for eligibility, and 67 were excluded for the following reasons: 23 were on adult subjects, 15 did not provide extractable data on pediatric subjects, 12 were reviews, 6 were not in English, 5 involved systemically healthy pediatric subjects, 2 were in vitro studies, 2 did not assess saliva/supra-subgingival samples, 1 did not evaluate HHV microbiological content, and 1 did not focus on gingivitis/periodontitis.

Ultimately, eight records [34,35,36,37,38,39,40,41] met the eligibility criteria for inclusion in this systematic review prior to the manual search.

A manual search was conducted using the reference lists from the eight included studies [34,35,36,37,38,39,40,41] to identify any additional relevant articles. The manual search yielded 610 records, with 141 duplicates removed. After screening the remaining 469 titles and abstracts, 405 were considered irrelevant to the study topic. Of the 64 records evaluated for eligibility, full-text screening led to the exclusion of all 64 for the following reasons: 26 were on adult subjects, 20 did not assess HHV microbiological content, 8 did not evaluate saliva/subgingival microbial content, 6 were reviews, 2 did not allow for data extraction on pediatric subjects, 1 was not focused on gingivitis/periodontitis, and 1 involved systemically healthy pediatric subjects.

No additional records meeting the eligibility criteria were identified through the manual search.

Thus, the present systematic review includes eight articles [34,35,36,37,38,39,40,41] that examined the microbiological sampling of HHV, fungi, and periodontal pathogens in subgingival and/or saliva samples from systemically compromised pediatric subjects with gingivitis or periodontitis.

**Figure 2:**
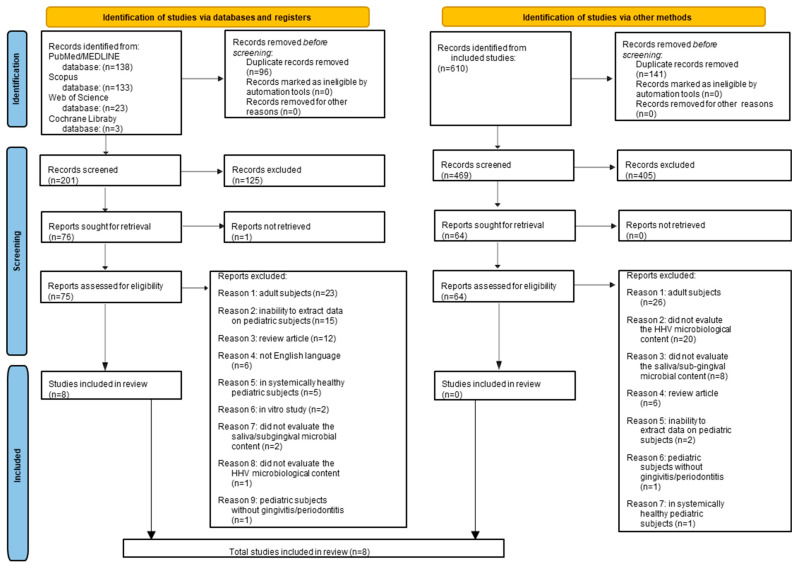
PRISMA 2020 flowchart for the study selection of systematic reviews.

Figure 2 presents the PRISMA 2020 flowchart for the study selection of electronic and manual searches.

Data from the eight studies [34,35,36,37,38,39,40,41] were collected regarding microbiological sampling of HHV, fungi, and periodontal pathogens in subgingival and/or salivary samples from systemically compromised pediatric subjects (≤18 years of age) with gingivitis or periodontitis. Table 3 outlines the characteristics of the gingivitis/periodontitis group and clinically healthy periodontium group, including population characteristics, periodontal parameters, and microbiological analysis characteristics. Table 4 details the microbiological sampling (HHV, periodontal pathogens, and fungi) of the gingivitis/periodontitis and clinically healthy periodontium groups.

Of the eight studies included in the present systematic review [34,35,36,37,38,39,40,41], four were case reports [34,37,39,40], three were case–control studies [35,36,38], and one was a case series [41].

Data from subjects with gingivitis and/or periodontitis were extracted exclusively if they satisfied the predefined eligibility criteria, excluding information related to other oral pathologies or non-pediatric populations. Data from pediatric subjects exhibiting a clinically healthy periodontal status were included for comparative purposes in accordance with the PICO model criteria. To ensure methodological consistency, definitions of periodontal conditions were standardized based on the 2017 classification system established by the AAP and EFB [1], thereby facilitating the alignment of studies conducted prior to this classification with the updated diagnostic framework.

### 3.1. Systemically Compromised Pediatric Subjects with Gingivitis and/or Periodontitis

#### 3.1.1. Population Characteristics

Sixty-nine systemically compromised pediatric subjects (56.56% of the total population examined in the studies included) [34,35,36,37,38,39,40] were diagnosed with periodontal disease. Among these, 35 (50.72%) systemically compromised pediatric subjects [38] were diagnosed with gingivitis, 22 with necrotizing gingivitis (32.88%) [35], and 12 with periodontitis (17.4%) [34,36,37,39,40,41].

The mean age was reported in seven studies [34,35,36,37,39,40,41], which was 9.78 ± 4.41 and ranged from 3 [41] to 17 years old [34,36]. Five studies [34,37,39,40,41] reported the gender ratio, which included three males [37,39,41] and three females [34,40,41] (1:1).

The country of origin was reported by two studies [34,35], which was Nigeria (n = 22) [35] and Lebanon (n = 1) [34]. The ethnicity was reported by one study [37], which was Caucasian (n = 1) [37].

The systemic diseases/syndromes reported by eight studies [34,35,36,37,38,39,40,41] were Acquired Immunodeficiency Syndrome (n = 35) [38], malnutrition/marasmic–kwashiorkor (n = 22) [35], Down syndrome (n = 6) [36], Kostmann syndrome (n = 2) [41], Dedicator of Cytokinesis 8 (DOCK8) deficiency (n = 1) [34], Hydroa Vacciniforme (n = 1) [39], Papillon–Lefèvre syndrome (n = 1) [40], and Fanconi anemia and diabetes mellitus (n = 1) [37].

The history of infectious diseases was reported in three studies [34,37,38,39] (accounting for 38 subjects), being negative in 1 subject and positive in 37 subjects [34,37,38], specifically for HIV (n = 35) [38], recurrent herpes labialis (n = 1) [37], and HSV-1 (n = 1) [39]. The history of infectious diseases was not defined for 31 subjects [35,36,40,41]. Previous pharmacological therapy for infectious diseases was reported in one study [34], with one subject administered Valacyclovir for Human Simplex Virus type-I-(HSV-I) [34].

#### 3.1.2. Periodontal Status

Gingivitis was diagnosed in 28.69% [38] of the total population of systemically compromised pediatric subjects examined in the included studies, while necrotizing gingivitis was diagnosed in 18.03% [35] and periodontitis in 9.84% [34,36,37,39,40,41].

Three studies [36,38,40] reported clinical parameters about systemically compromised pediatric subjects. In particular, PPD was evaluated by two studies in seven subjects with periodontitis [36,40] as follows: ≥5 mm (n = 6) [36]; from 6 to 9 mm (n = 1). Two studies [36,38] reported clinical parameters on bleeding in 41 subjects, in particular, 35 with gingivitis [38] and 6 with periodontitis [36]. The parameters evaluated were BoP, which was positive in 35 subjects (n = 35 gingivitis), and the Full Mouth Bleeding Score (FMBS) from the Ainamo and Bay classification in 6 subjects with periodontitis [36], expressed by the mean percentage values of 29.45 ± 19.3% (n = 6 periodontitis).

Other periodontal clinical parameters evaluated were the Full Mouth Plaque Score (FMPS) expressed by O’Leary classification in percentage, in six subjects with periodontitis [36], which was 96.67 ± 6.83%, and the Simplified Calculus Index (CI-S), expressed as mean percentage values, in six subjects with periodontitis [36], which resulted of 0.29 ± 0.16 (n = 6 periodontitis) [36].

Radiographic parameters were evaluated by one study [36] in six subjects with periodontitis, expressed through bone loss as follows: ≤1/3 (n = 2, periodontitis), ≥1/3 and ≤1/2 (n = 3, periodontitis), and ≥1/2 (n = 1, periodontitis) [36].

Crevicular parameters were not assessed by any of the studies included in the present systematic review.

#### 3.1.3. Microbiological Analyses

A total of 111 microbiological samples [34,35,36,37,38,39,40,41] were collected from the gingivitis/periodontitis sites, and the type of samples collected were 72 subgingival [34,35,36,37,39,40,41] and 39 saliva [34,38,39,41].

The sampling collection methods employed were sterile paper points in 68 [35,36,37,40], sterile containers in 35 [38], and sterile toothbrushes in 4 [41].

No periodontal treatment was performed prior to sample collection in any of the studies [34,35,36,37,38,39,40,41].

Microorganism identification techniques included PCR (n = 38) [34,38,41], nested PCR (n = 31) [35,36,37,39,40], and culture (n = 3) [34,37,40].

The microbial targets analyzed through the microorganism identification techniques were IE gene (n = 29) [35,36,40], EBNA1 gene (n = 22) [35], nucleocapsid protein gene (n = 22) [35], L1 gene (n = 22) [35], EBNA2 gene (n = 7) [36,40], and 16S rRNA (n = 1) [40].

The sampled sites reported were varied, with necrotizing gingivitis sites for 22 subjects [35], periodontitis sites for 8 subjects [36,37,39], and the deepest periodontitis sites in 1 subject [40].

#### 3.1.4. Bacterial Profile

Among the red complex bacteria, *Porphyromonas gingivalis* was investigated by two studies [34,41] in three subjects (4.37% of the total population) with periodontitis. In particular, one study [34] reported culture as an identification technique in one subject, which was positive (n = 1 periodontitis) [34], and one study [41] used PCR, which was negative in two subjects (n = 2 periodontitis) [41]. Among the three subjects investigated for *Porphyromonas gingivalis*, one individual (n = 1, 33.33% periodontitis) was positive [34] and two (n = 2, 66.67% periodontitis) were negative [41].

*Treponema denticola* was investigated in one study [34] in one periodontitis subject (1.45% of the total population) through culture, which resulted positive (n = 1, 100% periodontitis) [34].

*Tannerella forsythia* was sought across two studies [40,41] in three subjects (4.37% of the total population) with periodontitis. In particular, one study reported both the use of cultures and PCR as identification methods, of which the former was negative and the latter was positive (n = 1 periodontitis) [40]. One study [41] reported the use of PCR, which resulted negative in two subjects (n = 2 periodontitis) [41]. Among the three subjects investigated for *Treponema denticola*, a total of one individual (n = 1, 33.33% periodontitis) was positive [40], and two (n = 2, 66.67%, periodontitis) individuals were negative [41].

Among orange complex bacteria, *Prevotella intermedia*/*nigriscens* was investigated by two studies [40,41] in three subjects (4.37% of the total population) with periodontitis. In particular, one study [40] reported both the use of culture and PCR, which were positive in one subject (n = 1 periodontitis) [40], and the culture counts, expressed as a percentage of total culture, was 16.40% [40]. One study investigated bacteria content with PCR [41], which resulted positive in one subject (n = 1 periodontitis) [41]. A total of two subjects (n = 2, 66.67% periodontitis) [40,41] were positive for *Prevotella intermedia*/*nigriscens*, and one subject (n = 1, 33.33% periodontitis) was negative [41].

*Fusobacterium species* were investigated by one study [37] in one subject (1.45% of the total population) with periodontitis by culture. Specifically, it was found to be positive in one subject (n = 1, 100% periodontitis) [37], with a count expressed as a percentage of the total bacterial count of 2.2% [37].

*Fusobacterium nucleatum* was assessed by one study [40] in one subject (1.45% of the total population) with periodontitis through culture [40]. One subject (n = 1, 100% periodontitis) resulted positive at culture, with a count of 14.30% of total culture counts [40].

*Campylobacter species* were investigated by two studies [37,40] in two subjects (2.90% of the total population) with periodontitis through culture in one subject [37] and both culture and PCR in one subject [40]. One subject (n = 1 periodontitis) [37] was a positive at culture, with a count expressed as a percentage of the total culture count of 2.2% [37]. One subject (n = 1 periodontitis) resulted negative at both culture and PCR [40]. Among the two subjects investigated for *Campylobacter species*, a total of one individual (n = 1, 50% periodontitis) was positive [37], and one (n = 1, 50% periodontitis) individual was negative [40].

*Campylobacter rectus* was evaluated by one study [41] in two subjects (2.90% of the total population) with periodontitis via PCR analysis, which identified two positive subjects (n = 2, 100% periodontitis) [41].

*Parvimonas micra* was investigated by two studies [37,40] in two subjects (2.90% of the total population) with periodontitis through culture [37,40]. Specifically, two subjects (n = 2, 100% periodontitis) resulted positive [37,40], with a count, expressed as a percentage of total bacteria counts, of 3.4% [37] and 10.6% [40], respectively.

*Streptococcus constellatus* was investigated by one study [34] in one subject (1.45% of the total population) with periodontitis through bacterial culture, finding positivity in one subject (n = 1, 100% periodontitis), with a count expressed as a percentage of 2.2% of total the culture count [34].

*Eubacterium species* were investigated by one study [40] in one subject (1.45% of the total population) with periodontitis by culture [40], which was negative (n = 1, 100% periodontitis) [40].

Among yellow complex bacteria, *Streptococcus oralis* was investigated in one study [34] by culture in one subject (1.45% of the total population) with periodontitis, who resulted positive (n = 1, 100% periodontitis) [34].

In green complex bacteria, *Eikenella corrodens* was investigated by one study [40] in one subject (1.45% of the total population) with periodontitis, analyzed through both culture and PCR. Positivity was found in both (n = 1, 100% periodontitis) [40], with a culture count expressed as a percentage of 0.80% of the total culture counts [40].

*Capnocytophaga gingivalis* was investigated by one study [34] in one subject (1.45% of the total population) with periodontitis through culture, which was positive (n = 1, 100% periodontitis) [34].

*Capnocytophaga sputigena* was investigated by one study [34] in one subject (1.45% of the total population) with periodontitis by culture, which was positive (n = 1, 100% periodontitis) [34].

*Capnocytophaga ochracea* was investigated by one study [34] in one subject (1.45% of the total population) with periodontitis via culture, which resulted positive (n = 1, 100% periodontitis) [34].

*Camphylobacter concisus* was investigated by one study [34] in one subject (1.45% of the total population) with periodontitis by culture, which was positive (n = 1, 100% periodontitis) [34].

Among blue complex bacteria, *Actinomyces georgiae* was investigated by one study [34] in one subject (1.45% of the total population) with periodontitis by culture analysis, which resulted positive (n = 1, 100% periodontitis) [34].

None of the studies investigated bacteria belonging to the purple complex.

Of the bacteria outliers from the bacterial complexes, *Aggregatibacter actinomycetemcomitans* was investigated by three studies [37,40,41] in four subjects (5.80% of the total population) with periodontitis using culture (n = 1 periodontitis) [37], both culture and PCR (n = 1 periodontitis) [40], and PCR (n = 2 periodontitis) [41]. One subject (n = 1 periodontitis) [37] was positive at culture, with a count of 1.1% of the total culture counts; one was positive at both culture and PCR (n = 1 periodontitis) [40], with a culture count of 0.80% of the total bacterial count; and two were negative at PCR (n = 2 periodontitis) [41]. Among the four subjects investigated for *Aggregatibacter actinomycetemcomitans*, a total of two individuals (n = 2, 50% periodontitis) were positive [37,40], and two individuals (n = 2, 50% periodontitis) were negative [41].

*Enteric Gram rods* were investigated by one study [40], through culture, in one subject with periodontitis, who was negative (n = 1 periodontitis) [40].

*Β-hemolytic streptococci* were investigated by one study [40] in one subject (1.45% of the total population) with periodontitis, who was negative (n = 1, 100% periodontitis) [40].

None of the studies reported the total bacterial count.

#### 3.1.5. Viral Profile

Human Simplex Virus (HSV) was examined in 30 individuals (43.48% of the total population) across four studies [34,35,36,37]. Among them, 8 individuals had periodontitis (26.67%) [34,36,37], and 22 had necrotizing gingivitis (73.33%) [35]. Of the 30 individuals tested for HSV, 8 were positive (26.67%) (n = 5, 16.67% necrotizing gingivitis; n = 3, 10% periodontitis) [34,36,37], and 22 were negative (n = 17, 56.66% necrotizing gingivitis; n = 5, 16.67% periodontitis) [35,36].

HSV-I was assessed in 35 subjects (50.72% of the total population) with gingivitis in one study [38]. Among them, 3 subjects (n = 3, 8.57% gingivitis) were positive, and 32 were negative (n = 32, 91.43% gingivitis) [38].

Human Simplex Virus type II (HSV-II) was analyzed in 35 subjects (50.72% of the total population) with gingivitis in one study [38]. Among them, 2 subjects (n = 2, 5.72% gingivitis) were positive, and 33 were negative (n = 33, 94.28% gingivitis) [38].

Cytomegalovirus (CMV) was investigated in 67 subjects (97.10% of the total population) across six studies [35,36,37,38,40,41], of which 35 had gingivitis (52.24%) [38], 22 had necrotizing gingivitis (32.84%) [35], and 10 had periodontitis (14.92%) [36,37,40,41]. Among them, 22 were positive for CMV (32.83%) (n = 13,19.40% necrotizing gingivitis; n = 5, 7.46% gingivitis; n = 4, 5.97% periodontitis) [35,36,37,38,40], and 45 were negative (67.16%) (n = 30, 44.78% gingivitis; n = 9, 13.43% necrotizing gingivitis; n = 6, 8.96% periodontitis) [35,36,41].

Epstein–Barr virus (EBV) was examined in 39 subjects (56.52% of the total population) in four studies [34,38,39,41]; precisely, 35 subjects with gingivitis (n = 35, 89.74% gingivitis) [38] and 4 with periodontitis (n = 4, 10.26% periodontitis) [34,39,41]. EBV was positive in 3 subjects (n = 3, 7.69% periodontitis) [39,41] and negative in 36 (92.31%) (n = 1, 2.56% periodontitis, n = 35, 89.75% gingivitis) [38,41].

Epstein–Barr virus type I (EBV-I) was investigated in 29 subjects (42.03% of the total population) by three studies [35,36,40]; in particular, 22 individuals (75.86%) with necrotizing gingivitis [35] and 7 (24.14%) with periodontitis [36,40]. EBV-I was positive in 9 subjects (31.04%) (n = 6, 20.69% necrotizing gingivitis; n = 3, 10.34% periodontitis) [35,36,40] and negative in 20 (68.96%) (n = 16, 55.18% necrotizing gingivitis; n = 4, 13.79% periodontitis) [35,36].

Human herpesvirus 6 (HHV-6) was investigated in 23 subjects (33.33% of the total population) across two studies [35,39]; specifically, 22 (95.65%) subjects necrotizing gingivitis [35] and 1 (4.35%) with periodontitis [39]. Among them, 2 subjects (8.69%) were positive (n = 1, 4.35% necrotizing gingivitis; n = 1, 4.35% periodontitis) [35,39], and 21 were negative (91.30%) (n = 21, 91.30% necrotizing gingivitis) [35].

Human herpesvirus 7 (HHV-7) was evaluated in a single individual (n = 1, 1.45% of the total population) with periodontitis in one study [39], who resulted positive (n = 1, 100% periodontitis) [39].

Varicella-Zoster virus (VZV) was assessed in 35 subjects (50.72% of the total population) with gingivitis in one study [38], who resulted negative (n = 35, 100% gingivitis) [38].

Co-infections involving human herpesviruses (HHVs) were analyzed in 28 subjects (40.58% of the total population) across two studies [35,36], including 22 subjects (78.57%) with necrotizing gingivitis [35] and 6 (21.43%) with periodontitis [36]. Specifically, CMV and HSV co-infection were investigated in six subjects with periodontitis (21.43%); among them, one subject tested positive (n = 1, 16.67% periodontitis), and five subjects tested negative (n = 5, 83.33% periodontitis) [36]. Co-infections of three not-defined HHVs were investigated among 22 subjects (78.57%) with necrotizing gingivitis; among them, 6 subjects were positive (n = 6, 27.27%, necrotizing gingivitis), and 16 were negative (n = 16, 72.73% necrotizing gingivitis) [35]. Co-infections of two not-defined HHVs were investigated among 22 subjects (78.57%); among them, 2 (n = 2, 9.09% necrotizing gingivitis) were positive, and 20 were negative (n = 20, 90.91% necrotizing gingivitis) [35].

Other viruses beyond HHVs were investigated in 23 subjects (33.33% of the total population) across two studies [35,39]. In particular, Human Papilloma Virus (HPV) was investigated in 22 necrotizing gingivitis subjects [35], all of whom were negative (n = 22, 100% necrotizing gingivitis), and Human Parvovirus B-19 (HPV-B19) was investigated in 1 periodontitis subject, who tested negative (n = 1, 100% periodontitis) [39].

**Figure 3:**
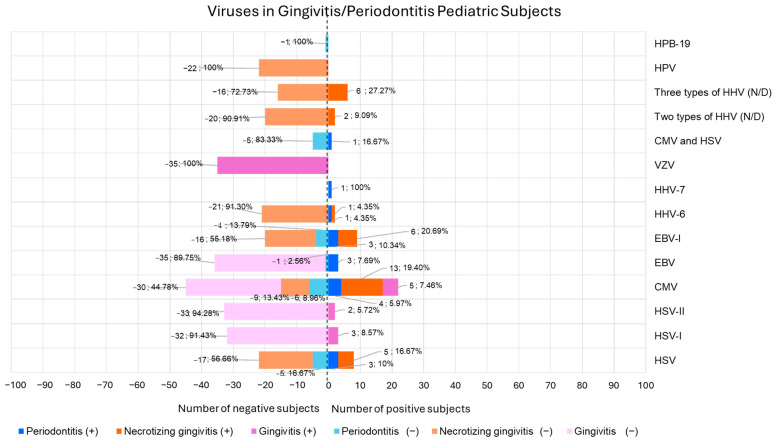
Viruses in gingivitis and periodontitis in systemically compromised pediatric subjects: number and percentage of negative (**left**) and positive (**right**) cases tested.

Figure 3 depicts the viruses in the gingivitis/periodontitis pediatric subjects.

#### 3.1.6. Fungal Profile

The fungal profile in systemically compromised pediatric subjects was investigated in three studies [34,37,40] in three subjects (4.35% of the total population) with periodontitis through culture. In particular, one subject (n = 1, 33.33% periodontitis) [37] was positive at fungi culture, reporting the presence of *Candida Albicans*, with a count expressed as a percentage of total culture counts of 0.3%. In contrast, fungi were not present in two subjects (n = 2, 66.67% periodontitis) [34,40].

### 3.2. Systemically Compromised Pediatric Subjects with a Clinically Healthy Periodontium

#### 3.2.1. Population Characteristics

In pediatric subjects with clinically healthy periodontium, the population consisted of 53 subjects (43.44% of the total population examined in the studies included) [35,38,40]. The mean age was reported by one study [35], which was 7.1 ± 1.4 years old, ranging from 3 to 14 years of age [35]. The gender ratio was not defined by any of the studies investigating pediatric subjects with clinically healthy periodontium.

The country of origin was reported by one study [35], which was Nigeria (n = 40). Two studies [35,38] reported comorbidities in 43 subjects, specifically, malnutrition (n = 40) [35] and Acquired Immunodeficiency Syndrome (n = 13) [38].

The history of infectious disease was reported by one study [38] in 13 pediatric subjects with HIV. None of the studies defined the history of pharmacological therapy.

The periodontal conditions reported were clinically healthy periodontium (n = 53) [35,38,40].

#### 3.2.2. Periodontal Status

None of the studies investigating pediatric subjects with clinically healthy periodontium reported clinical, radiographic, or crevicular periodontal parameters.

#### 3.2.3. Microbiological Analyses

A total of 93 microbiological samples [35,38] were collected from clinically healthy periodontium pediatric subjects, and the type of sample collected was 80 subgingival [35] and 13 salivary [38].

The sampling collection methods employed were sterile paper points (n = 83) [35,40] and sterile containers (n = 13) [38].

No periodontal treatment was performed prior to sample collection in any of the studies investigating pediatric subjects with clinically healthy periodontium [35,38].

The microorganism identification techniques included nested PCR (n = 80) [35] and PCR (n = 13) [38].

The microbial targets analyzed through the microorganism identification technique were the IE gene (n = 80) [35], EBNA1 gene (n = 80) [35], gag gene (n = 80) [35], and nucleocapsid protein gene (n = 80) [35].

#### 3.2.4. Bacterial Profile

None of the studies investigating pediatric subjects with clinically healthy periodontium reported bacterial profiles in the systemically compromised pediatric subjects.

#### 3.2.5. Viral Profile

HSV was examined in 40 individuals (74.07% of the total population with clinically healthy periodontium) across one study [35]. Among them, 1 individual (n = 1, 2.50%) was positive [35], and 39 subjects (n = 39, 97.5%) were negative [35].

HSV-I was assessed in 13 subjects (24.07% of the total population with clinically healthy periodontium) in one study [38]. Among them, 13 subjects (n = 13, 100%) resulted negative [38].

HSV-II was analyzed in 13 subjects (24.07% of the total population with clinically healthy periodontium) in one study [38]. Among them, 13 subjects (n = 13, 100%) resulted negative [38].

CMV was investigated in 53 subjects (100% of the population with clinically healthy periodontium) across two studies [35,38], of which 8 subjects were positive (n = 8, 15.09%) [35,38], and 45 (n = 45, 84.91%) were negative [35,38].

EBV was examined in 13 subjects (24.07% of the total population with clinically healthy periodontium) in one study [38]. Among them, 13 subjects (n = 13, 100%) resulted negative [38].

EBV-I was examined in 40 individuals (75.47% of the total population with clinically healthy periodontium) across one study [35]. Among them, 2 subjects (n = 2, 5.00%) were positive [35], and 38 (n = 38, 95.00%) were negative [35].

HHV-6 was investigated in 40 individuals (74.07% of the total population with clinically healthy periodontium) across one study [35]. Among them, 40 (n = 40, 100%) were negative [35].

VZV was assessed in 13 subjects (24.07% of the total population with clinically healthy periodontium) in one study [38], who were negative (n = 13, 100%) [38].

Co-infections involving HHVs were analyzed in 40 subjects (74.07% of the total population with clinically healthy periodontium) across one study [35]. Specifically, co-infections of two not-defined HHVs were negative in 40 subjects (n = 40, 100%), and co-infections of three not-defined HHVs were negative in 40 subjects (n = 40, 100%) [35].

#### 3.2.6. Fungal Profile

None of the studies investigating pediatric subjects with clinically healthy periodontium reported fungal profiles.

### 3.3. Quality Assessment

Two studies [36,38] were judged to be at a low risk of bias, and one [35] was at a moderate risk of bias, through the use of the ROBINS-I tool, as reported in Table S1 in Supplementary File S1.

The four case report studies [34,37,39,40] were judged to be includible through the use of the JBI for case reports, as reported in Table S2 in Supplementary File S1.

One case series study [41] was judged to be includible through the use of the JBI for case series, as reported in Table S3 in Supplementary File S1.

## 4. Discussion

### 4.1. Population Characteristics

Among the 122 pediatric subjects examined in the included studies, 69 (56.56%) systemically compromised individuals were diagnosed with periodontal disease, while 53 (43.44%) had a clinically healthy periodontium.

Gingivitis was the most frequently reported condition (n = 35, 50.72%), followed by necrotizing gingivitis (n = 22, 32.88%) and periodontitis (n = 12, 17.4%).

In the subjects with periodontal disease, the mean age across seven studies was 9.78 ± 4.41 years, ranging from 3 to 17 years, whereas, in those with a healthy periodontium, the mean age was reported in one study as 7.1 ± 1.4 years, ranging from 3 to 14 years. Although age differences were observed, the small sample sizes may limit considerations regarding age-related susceptibility, despite the potential influence of age on periodontal disease susceptibility [42].

The country of origin reported for the subjects with periodontal disease was Nigeria in twenty-two subjects and Lebanon in one. In contrast, for those with a healthy periodontium, Nigeria was the country of origin for 40 subjects, while the Caucasian ethnicity was reported in one subject with periodontal disease. The limited geographical representation may have influenced the periodontal status, microbiological profiles, and immune responses to the oral microbiome components. Indeed, variations in oral microbial composition and immune responses across regions could contribute to differences in periodontal disease prevalence and severity [43,44]. Additionally, socioeconomic factors, nutritional status, and access to dental care may further modulate disease susceptibility [45,46,47].

Comorbidities were widely varied among those with periodontal disease, with the most frequently reported conditions being HIV/AIDS (n = 35), malnutrition-related disorders (n = 22), and congenital syndromes (n = 12). In contrast, comorbidities were documented in 43 subjects with a healthy periodontium, predominantly malnutrition (n = 40) and HIV/AIDS (n = 13). The predominance of immunocompromised conditions may highlight the association between systemic health and periodontal status. Immunosuppressed individuals are more susceptible to microbial dysbiosis and exaggerated inflammatory responses, which may lead to severe periodontal breakdown [31,48,49]. Notably, malnourished children were present in both groups, suggesting that malnutrition alone might not necessarily result in periodontal disease, but its interaction with other systemic and local factors may exacerbate susceptibility.

A history of infectious diseases was reported in 38 subjects with periodontal disease, primarily HIV, but also HSV-1 and recurrent herpes labialis. In comparison, a history of infectious diseases was documented in 13 subjects with a healthy periodontium, all of whom were HIV-positive.

Pharmacological history was limitedly documented, with one subject with periodontal disease receiving Valacyclovir for HSV-1, while none of the studies reported pharmacological history in the subjects with a healthy periodontium. The absence of data on medication use may have limited the ability to evaluate the impact of systemic drugs or other treatments on periodontal outcomes.

### 4.2. Periodontal Status in Systemically Compromised Pediatric Subjects

Periodontal disease was diagnosed in 69 systemically compromised pediatric subjects (56.56% of the total population), with 12 cases of periodontitis, mostly generalized. In contrast, the systemically healthy pediatric subjects were frequently diagnosed with molar–incisor pattern periodontitis (MIPP) [50]. The predominance of generalized forms in the compromised subjects suggests that underlying systemic conditions may have contributed to a more widespread periodontal involvement, whereas the systemically healthy children, likely with a more effective immune response, exhibited more localized patterns.

Periodontal health was observed in 43.44% of the total subjects, compared to 56.50% of the systemically healthy children [50]. These findings underlined a mirrored trend as periodontal health status was more common among the systemically healthy children, whereas in the systemically compromised children, systemic conditions may have increased susceptibility to periodontal disease.

**Figure 4:**
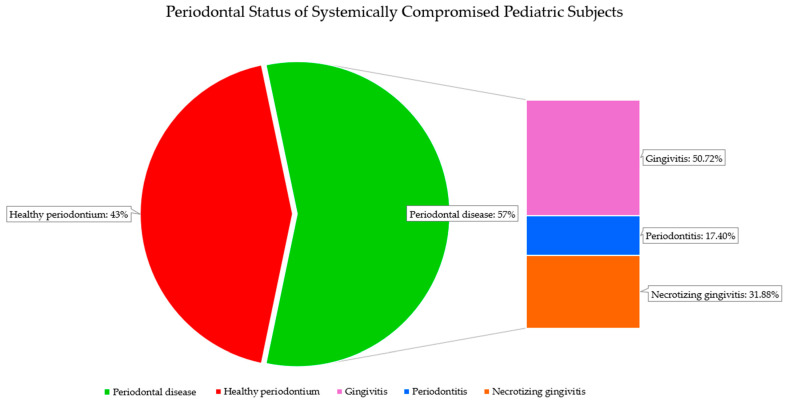
Periodontal status in systemically compromised pediatric subjects.

Among the 122 total subjects, 53 (43.44%) were periodontally healthy, while 69 (56.56%) had periodontal conditions, including gingivitis (35 cases, 50.72%), necrotizing gingivitis (22 cases, 31.88%), and periodontitis (12 cases, 17.40%). Notably, necrotizing gingivitis was found in the systemically compromised children, whereas it was absent in the systemically healthy subjects [50], suggesting systemic diseases may heighten the risk of developing periodontal conditions and drive more severe forms, possibly due to impaired immune regulation and an intensified inflammatory response, ultimately exacerbating tissue damage. Figure 4 shows the periodontal status of the systemically compromised pediatric subjects.

Although the assessment of periodontal and radiographic parameters was limited across the included studies, and crevicular parameters were not investigated, the reported data may contribute to characterizing the periodontal status of systemically compromised pediatric subjects.

Bleeding on Probing was positive in all of the 35 subjects with gingivitis [38].

An FMBS of 29.45 ± 19.3% and FMPS of 96.67 ± 6.83% were registered in the six subjects with periodontitis and Trisomy 21, who generally exhibited PPD values ≥5 mm and alveolar bone loss ≤1/3. Three subjects had bone loss between one-third and one-half, and one subject had bone loss ≥1/2 [36]. These parameters align with expectations since all six pediatric subjects studied were affected by Trisomy 21, where periodontitis prevalence is notably higher compared to the general population [29,51]. This is likely due to compromised host defenses, including a reduction in neutrophils, monocytes, and T lymphocytes [29,52]. Furthermore, individuals with Down syndrome often present with microdontia, short roots, and mucogingival issues, all of which contribute to an increased risk of developing periodontitis [51,53].

Additionally, both short roots and taurodontism may reduce the effectiveness of periodontal attachment, leading to tooth mobility and further contributing to the development of periodontal disease [51,53]. Moreover, patients with Down syndrome exhibit lower salivary flow compared to healthy individuals [54,55] due to factors such as open-mouth posture, protruding tongue, hypotonic orofacial muscles, and drooling, which reduce stimulated salivary flow from the parotid gland [55,56]. The diminished salivary flow might have failed to dilute and eliminate bacterial cells and metabolites adequately [54,55], potentially underlying, at least in part, the high FMPS detected.

### 4.3. Bacterial Profile in Systemically Compromised Pediatric Subjects

#### 4.3.1. Bacteria in Pediatric Subjects with Gingivitis/Periodontitis

Although the characterization of the bacterial profile in systemically compromised pediatric subjects was not extensively investigated, data were retrieved from individuals with periodontitis; conversely, no data were found concerning the bacterial profile in gingivitis or necrotizing gingivitis subjects.

*Porphyromonas gingivalis* was detected in 33.33% of the tested individuals. Interestingly, the bacterium was found in one subject with DOCK8 deficiency, a condition characterized by immunodeficiency affecting both humoral and cellular immunity [57]. The systemic immune dysfunction in DOCK8 deficiency may create a permissive environment for the colonization of anaerobic Gram-negative bacteria, which evade host immune responses through phagocytosis inhibition [14,58,59,60]. Indeed, other bacterial species associated with this condition, such as *Treponema denticola* and members of the green and yellow complexes, including *Capnocytophaga* species (*C. gingivalis*, *sputigena*, *ochracea*), may have contributed to disease progression.

Intriguingly, despite its frequent presence in periodontitis, *Porphyromonas gingivalis* was absent in the subjects affected by Kostmann syndrome. The immunological landscape in Kostmann syndrome, marked by severe neutropenia and defects in bacterial clearance mechanisms, might favor the proliferation of other pathogenic species [61]. Notably, Prevotella intermedia was detected in this cohort, mainly associated with Kostmann syndrome and Papillon–Lefèvre syndrome. *Prevotella intermedia*/*nigriscens* is characterized by the ability to withstand oxidative stress and establish robust biofilms, particularly in environments with low neutrophil activity, which may have provided its selective advantage in these patients [61,62,63].

The presence of *Aggregatibacter actinomycetemcomitans* in the Fanconi anemia and diabetes mellitus subjects may be attributed to the altered metabolic environment, particularly changes in glucose metabolism that may have favored its colonization and persistence [14,20,64]. Conversely, *Aggregatibacter actinomycetemcomitans* was absent in the pediatric subjects with Kostmann syndrome. The bacteria may have a more pronounced ability to exploit metabolic dysregulation rather than relying on immune evasion. Therefore, it may be likely that neutropenia in Kostmann syndrome may not create an optimal niche for this bacterium, which is thereby absent in these individuals.

However, the presence of keystone pathogens associated with periodontitis may not solely justify the onset of the disease, just as the absence of bacteria commonly linked to periodontitis may not exclude disease manifestation [11,65]. Indeed, periodontal disease is a multifactorial condition influenced by the complex interplay between bacterial communities, viral and fungal components of the microbiome, and, most importantly, the host immune response [1,11,14]. The immune status of the individuals, particularly in the context of systemic diseases, may critically shape the microbiological profile and determine the progression and severity of periodontal disease.

A comparison of the bacterial profiles between systemically compromised pediatric subjects and those systemically healthy, as investigated in a previous systematic review [50], may reveal relevant insight, particularly in the context of periodontitis. Indeed, although the current study had a more limited sample size compared to that of the systemically healthy pediatric subjects, both groups shared notable bacterial patterns, particularly within the red (e.g., *Porphyromonas gingivalis* and *Tannerella forsythia*) and orange (e.g., *Prevotella intermedia*/*nigriscens* and *Fusobacterium nucleatum*) complexes, which are commonly found in periodontitis [50]. This finding underscored a remarkable constancy in the microbial constituents involved in the pathogenesis of periodontitis, which could be independent of systemic health status.

Notably, a divergence emerged in the systemically compromised individuals, as a more constrained bacterial profile, with a reduced presence of bacterial species and a diminished variety, was found, suggesting the potential influence of an impaired immune system on the oral microbiome composition. This reduction in bacterial diversity may reflect a dysregulated immune response or a diminished ability to maintain a balanced ecological equilibrium among oral bacteria. In contrast, the systemically healthy subjects displayed a broader and more diversified bacterial profile, characterized by a wider variety of species, which may be due to a proper immune system function [50].

Furthermore, pediatric subjects with systemic diseases may harbor opportunistic species or bacteria associated with systemic complications, which are less frequently observed in the systemically healthy group [31,63,66]. Accordingly, it may be supposed that the diminished immune capacity in these individuals may favor the colonization of bacteria that would otherwise not be predominant, further complicating the oral microbial environment. This could lead to more severe periodontal conditions, where a less diverse but potentially more virulent microbiome might be prevalent.

#### 4.3.2. Bacteria in Pediatric Subjects with Healthy Periodontium

Although plaque accumulation in pediatric subjects is a predisposing cofactor for gingivitis and periodontitis, the included studies did not provide data on the Plaque Index (PI), Full Mouth Plaque Score (FMPS), Bleeding on Probing (BOP), or radiographic and crevicular parameters. Therefore, it was not possible to define the hygiene status of the examined subjects.

Nevertheless, it could be hypothesized that these subjects maintained effective biofilm control, preventing its organization toward dysbiosis. Furthermore, considering that the bacterial profile was not investigated, it might also be plausible that despite underlying systemic conditions, the immune system successfully compensated for potential imbalances, preserving periodontal health status through an adequate modulation of the inflammatory response.

In conclusion, the bacterial profile in the systemically compromised pediatric patients with periodontitis varied based on the underlying condition. *Porphyromonas gingivalis* was found in those with DOCK8 deficiency, while Prevotella intermedia was prevalent in Kostmann syndrome. *Aggregatibacter actinomycetemcomitans* appeared in the patients with Fanconi anemia and diabetes, indicating that metabolic factors may favor its colonization. Although there were some similarities with the systemically healthy children with gingivitis and periodontitis regarding red and orange complex bacteria, the compromised individuals showed reduced bacterial diversity due to immune dysfunction. This limited variety, along with potential opportunistic species, likely leads to a more dysregulated periodontal environment. There were no specific bacterial data for systemically compromised children with healthy periodontium, but effective immune regulation and biofilm control likely contributed to their periodontal health.

### 4.4. Viral Profile in Systemically Compromised Pediatric Subjects

#### 4.4.1. Viruses in Gingivitis and/or Periodontitis Pediatric Subjects

VZV was not detected in any cases, consistent with its preference for skin epithelium and vesicular rash presentation over oral mucosa involvement compared to HSV exhibiting; instead, there was a stronger tropism for oral epithelial cells [21,22,67], especially in gingivitis cases. In periodontitis, CMV and EBV were more frequently identified, with CMV detected in 5.97% and EBV in 7.69% of the tested subjects, where EBV-I showed the highest prevalence (10.34%).

HSV-I and HSV-II were more prevalent in gingivitis (8.57% and 5.72%, respectively) than in the other periodontal conditions. This may suggest that HSV infections may preferentially occur in conditions predominately affecting the superficial epithelial layers, which may be conducive to viral replication, as opposed to conditions like periodontitis that involve deeper tissue structures [67]. However, it is noteworthy that most of the gingivitis cases tested negative for both HSV-I and HSV-II, potentially due to the impact of systemic conditions like malnutrition/kwashiorkor. Indeed, this condition impairs epithelial turnover and compromises mucosal barrier integrity, potentially hindering viral replication [68]. The lack of essential nutrients for cellular regeneration may have limited the ability of HSVs to reactivate or replicate in these subjects, leading to the higher rate of negativity observed.

A similar trend was also observed in the pediatric subjects with necrotizing gingivitis, revealing a higher prevalence of HSV (16.67%) compared to those with periodontitis (10.00%).

Conversely, in periodontitis, CMV and EBV were more frequently identified. Specifically, among CMV-positive cases, one subject showed positivity to active CMV while testing negative for its latent form, suggesting that active infection may play a more direct role in modulating inflammation and accelerating disease progression compared to its latent presence. Active CMV amplifies the inflammatory response by producing pro-inflammatory cytokines, such as interleukin-1β and TNF-α, which promote tissue destruction and osteoclastogenesis [21].

Moreover, CMV may interact with periodontal bacterial species in biofilm, enhancing their pathogenic effects and contributing to microbial dysbiosis and impaired host defenses [69]. This interaction may likely explain why CMV was more frequently associated with advanced forms of periodontitis in the systemically compromised patients than HSV.

In gingivitis, CMV detection (19.40%) was linked to chronic immunosuppression, which potentially facilitates viral reactivation and reduces the ability to control infections or clear latent viruses [70,71,72].

Similarly, the identification of CMV in necrotizing gingivitis (19.40%) may reflect immune impairment due to malnutrition/kwashiorkor, where the essential nutrient deficiencies may have altered T and B lymphocyte function, diminishing the ability to mount an effective response against *Herpesviridae* infections.

EBV and EBV-I were mainly found in necrotizing gingivitis and periodontitis, indicating their potential association with these conditions.

However, the higher negative rates for EBV in necrotizing gingivitis and gingivitis than periodontitis may indicate a stronger tropism of EBV for deeper periodontal structures. This observation aligns with the ability of EBV to persist in pools of infected B-cells within the junctional and sulcular epithelium [73,74], potentially fostering its involvement in the progression of periodontitis rather than gingivitis.

Underlying immune dysfunction in conditions like Down syndrome, Papillon–Lefèvre syndrome, Kostmann syndrome, and Hydroa vacciniforme may have predisposed these individuals to higher EBV rates, as currently found, potentially fostering EBV-associated periodontal complications through distinct mechanisms.

For instance, in Down syndrome, altered T-cell subpopulations and increased matrix metalloproteinase activity promote excessive inflammation and tissue breakdown [26,51,75,76], which may facilitate EBV reactivation. Similarly, functional deficiencies in neutrophils and impaired antimicrobial defenses in Papillon–Lefèvre and Kostmann syndromes [26] may create an environment conducive to viral reactivation and persistent inflammation. In Hydroa vacciniforme, an EBV-associated T-cell lymphoproliferative disorder [77], EBV-infected macrophages within the periodontal tissues may trigger chronic inflammation, weakening periodontal integrity.

These findings may suggest that CMV and EBV may have a variable role in the pathogenesis of periodontitis, gingivitis, and necrotizing gingivitis in systemically compromised pediatric subjects. In periodontitis, these viruses may act as more pronounced cofactors, amplifying inflammation, immune activation, and tissue destruction by interacting with toll-like receptors prominently expressed in periodontal lesions [21,22,78].

In contrast, in gingivitis and necrotizing gingivitis, CMV and EBV may contribute more to amplifying inflammation in the context of systemic immune compromise, compounding the effects of microbial dysbiosis and impaired mucosal barrier function.

HHV-6 was detected in 4.35% of the tested subjects and was associated with necrotizing gingivitis and periodontitis cases. HHV-7 was detected in a periodontitis subject with Hydroa Vacciniforme. HHV-6 and HHV-7 have tropism for CD4+ T lymphocytes but can also infect monocytes, macrophages, and fibroblasts [21,79,80,81]. Their presence in gingival tissues suggests that the gingiva may serve as a reservoir for these viruses, which remain in latent form and can reactivate under conditions of immunosuppression [21,79,80,81]. The concomitant positivity for other HHVs, such as EBV and CMV in the specimens examined, might have facilitated the reactivation of HHV-6 and HHV-7 present in latent form by altering the local immune response. In the context of immune compromisation, secondary to underlying systemic diseases, this viral reactivation might have contributed to the pathogenesis of periodontitis and necrotizing gingivitis by promoting inflammation and tissue destruction.

Notably, in addition to HHVs, other viruses were investigated. This systematic review found that in cases of necrotizing gingivitis, the tested subjects were negative for HPV, and one subject with periodontitis tested negative for Human Parvovirus B-19. The absence of HPV detection was unexpected, especially considering the higher prevalence of oral HPV in certain regions, such as Nigeria, from which the subjects with kwashiorkor originated [82,83,84]. One possible explanation for these findings is viral interference, where certain viruses can inhibit the replication or detection of others by modulating the host’s immune response, potentially leading to competitive interactions among co-infecting viruses [85,86,87]. In these cases, it may be possible that the presence of HHVs competitively suppressed the replication of non-herpesviruses, like HPV and Parvovirus B-19.

Co-infections involving multiple HHVs were detected in 16.67% of the periodontitis cases, mainly CMV and HSV co-infection. Co-infections involving two or three unspecified HHVs were also detected in necrotizing gingivitis.

These findings suggest that under conditions of immune impairment caused by systemic diseases and local factors like bacterial dysbiosis, HHVs may coexist, utilizing host resources and potentially enhancing their persistence and replication. Studies have shown that microbial communities and viral co-infections can influence pathogens’ virulence and survival, supporting the concept of interdependent viral dynamics within the host [17,88].

The relationship between HHVs and clinical and radiographical periodontal parameters in the systemically compromised pediatric subjects was also noteworthy.

The presently found periodontal probing depths (PPDs) were suggestive of severe periodontal destruction, often observed in the pediatric subjects with systemic or genetic diseases [89,90,91]. Radiographic parameters also revealed bone loss ranging from ≤1/3 and ≥1/2 in periodontitis cases.

HHVs, like CMV and EBV, are linked to more severe clinical periodontal parameters and the dysregulation of the RANKL/OPG ratio, along with pathogen–bacterial interactions, potentially underscoring bone resorption [22,74,92,93]. These findings may explain the complexity of periodontal destruction, where the combination of several factors, including viral infections, virus–bacteria interactions, and immune system dysfunction in these patients, may accelerate the progression of periodontal disease.

Additionally, the reported clinical parameters related to inflammation, such as Bleeding on Probing (BoP+) and the Full Mouth Bleeding Score (FMBS), may further highlight the potential role of HHVs in tissue inflammation.

Emerging evidence suggests that microRNAs (miRNA) encoded by herpesviruses in human gingival tissue may alter the host cell transcriptome and modulate the immune response, contributing to chronic inflammation and tissue destruction [94]. Naqvi et al. [95] evidenced that microRNAs, as post-transcriptional regulators, are crucial for maintaining tissue homeostasis, but their aberrant expression is linked to several diseases, including periodontitis. MiRNAs produced by HHVs may alter immune responses in gingival tissue, impairing the balance between humoral and cellular host defenses against periodontal bacteria and viruses [95]. It might be likely that the mechanisms of tissue senescence and immune dysregulation, typical of the systemic compromisation of these individuals, may further be aggravated by subgingival and/or salivary HHV infections. HHV-produced miRNAs could hypothetically modulate the local immune cells, promoting a chronic pro-inflammatory environment. This phenomenon may have contributed to the observed clinical parameters (BoP+, FMBS) in the individuals with gingivitis and/or periodontitis and systemic diseases by accelerating periodontal tissue destruction and disease progression in immune-compromised contexts.

The Full Mouth Plaque Score (FMPS) values and Simplified Calculus Index (CI-S), found in the subjects with periodontitis and Down syndrome, could be attributed to difficulties related to oral hygiene management. The poor cooperation often observed in this population, related to cognitive and manual limitations, as well as dietary habits, makes learning and maintaining adequate oral hygiene techniques complex [29,52,96,97,98]. This condition may have favored the accumulation of plaque and mature bacterial biofilm, representing a protective microenvironment and a favorable substrate for colonization by HHVs, which may have been an aggravating factor in the impairment of periodontal health in the pediatric subjects.

The pediatric individuals with gingivitis and periodontitis, but otherwise systemically healthy, reported a higher prevalence of HHVs in periodontitis, with EBV being detected in 36.24% [50]. CMV was found in 50% of the cases, specifically 36.36% associated with periodontitis, and HSV was present in 23.53% of the cases, mostly in molar–incisor pattern periodontitis [50]. The immune system in non-compromised individuals might typically control viral infections, but persistent inflammation in periodontitis, especially in chronic forms, may allow for viral reactivation.

In contrast, the present study on systemically compromised individuals showed a different distribution of HHV prevalence. HSV was found to be positive in 26.67% of the cases, especially involving necrotizing gingivitis, while CMV was detected in 32.83% of the cases, primarily in necrotizing gingivitis and periodontitis. Interestingly, CMV and HSV were more prevalent in acute conditions like necrotizing gingivitis in the systemically compromised individuals. This might be attributed to their impaired immune response, which had likely facilitated viral reactivation.

These findings may indicate that while non-compromised individuals tend to have higher viral loads in chronic conditions like periodontitis, systemically compromised individuals show a greater prevalence of viral infections in acute conditions such as necrotizing gingivitis. In contrast, the inability to mount an effective immune response may lead to a higher viral burden in these severe, rapidly progressing diseases.

#### 4.4.2. Viruses in Clinical Healthy Periodontium

The presence of HHVs in the pediatric subjects with clinically healthy periodontium was observed, despite the majority of cases testing negative for these viruses. This phenomenon may be attributed to the ability of HHVs to persist in a latent state within oral tissues, serving as viral reservoirs without necessarily driving pathological processes [22,99]. HHVs are indeed known to cross barriers, exhibiting tissue-specific tropism, and establish infections that can remain silent for extended periods [21,88,100,101]. During latency, these viruses can evade the host immune system and reactivate under conditions of immune suppression or stress [23,102,103].

The lower prevalence or absence of HHVs in healthy periodontal tissues suggests that these viruses might exist in a latent, non-pathogenic state, coexisting within a balanced oral microbiome without contributing to disease progression [15,22,50]. This aligns with the findings of the present study, as most of the subjects with periodontally healthy status tested negative for HHVs, while a small proportion tested positive.

In particular, among the positive HHV cases, CMV and EBV-I were more frequently detected (15.09% and 5.00%, respectively). These findings may reflect not only viral latency but also the impact of systemic conditions on immune function, as HHV positivity was observed in the subjects affected by malnutrition/kwashiorkor and HIV.

In HIV-positive individuals, immune dysfunction—characterized by reduced CD4+ T-cells and immune activation—could permit persistent HHV infections, even in the absence of overt periodontal disease. In these cases, HHV may persist subclinically or transiently without sufficient local or systemic triggers to drive reactivation or cause tissue damage [71,104]. Furthermore, in subjects undergoing highly active antiretroviral therapy (HAART), HHV replication may remain low but stable, supported by improved immune function [105,106], potentially preventing periodontal disease despite viral persistence.

Similarly, in malnutrition/kwashiorkor, immune suppression may facilitate viral persistence. However, the absence of periodontal disease in these subjects may suggest that the viral load was insufficient to initiate pathologic processes, possibly due to a lack of other contributing factors, such as the presence of periodontal pathogenic bacteria.

In line with this, the predominance of the currently observed HHV-negative results might be explained by the absence of key cofactors, which typically foster co-infections and viral reactivation. Indeed, most of the pediatric subjects were negative for HSV, CMV, and EBV-I. Moreover, the absence of viral co-infections in the healthy periodontium subjects further supports the idea that a local microbial environment—comprising periodontal bacteria and fungi—may play a critical role in facilitating viral coexistence and triggering the host’s immune response, ultimately contributing to the onset of periodontal disease.

Without a well-organized biofilm, viruses may coexist in the periodontal area without triggering disease, as they alone are unlikely to initiate pathological processes de novo. They may act more as latent bystanders, potentially serving as cofactors for periodontal disease progression rather than as initiators. The lack of a favorable bacterial environment may prevent the viruses from exerting their destructive effects on the periodontium. Supporting this hypothesis, Slots et al. [21,23] suggest that virus–bacteria interactions might facilitate viral reactivation, contributing to disease progression only when microbial environmental conditions are conducive.

This suggests that HHVs may be present in a latent or subclinical form in individuals with clinically healthy periodontium and that their interaction with specific bacterial species, as well as other microorganisms in the microbiome, may influence their reactivation and contribute to disease progression under certain conditions. In the absence of a supportive microbial environment or co-infections, these viruses are unlikely to independently initiate disease processes but may act as cofactors when microbial conditions are favorable.

The viral profile in the pediatric subjects with and without systemic conditions, both exhibiting a clinically healthy periodontium, revealed distinct viral trends [50].

CMV was detected in 17.80% of the systemically healthy individuals [50] and 15.09% of those with systemic diseases, suggesting its persistence is independent of systemic status, likely influenced by localized immune surveillance or viral latency mechanisms.

Notably, EBV was predominantly negative in both cohorts, suggesting that its pathogenic potential may necessitate a conducive bacterial biofilm environment to exert a clinical impact. Indeed, the absence of periodontal pathogens like *Aggregatibacter actinomycetemcomitans* reported in the systemically healthy individuals could explain the limited pathogenic activity of HHVs, as this bacterium is linked to heightened susceptibility to viral reactivation [19,64,107].

EBV-I was more prevalent in the systemically healthy subjects (22.09%) [50] than in systemically affected ones (5.00%), potentially reflecting differential immune regulation. The underlying systemic conditions may either suppress viral replication or alter the periodontal environment by impeding EBV persistence. VZV was absent in both, potentially underscoring its lack of affinity for periodontal tissues.

No co-infections were detected in either cohort, suggesting that a stable microbiome associated with a healthy periodontium may not be conducive to the concurrent persistence of multiple herpesviruses. Accordingly, although crevicular parameters in the examined cohorts were not investigated, it might be plausible that crevicular fluid could have exerted a role in immune-mediated clearance by antimicrobial peptides and cytokines, which could have limited viral colonization [75,108,109]. Additionally, distinct metabolic requirements among herpesviruses might limit co-persistence due to competition for cellular resources [110,111].

Notably, HSV-I and HSV-II exhibited divergent detection patterns. In the systemically healthy children, HSV-I was detected in 100% of the tested subjects, whereas in the currently investigated systemically affected children, it was absent (0%). HSV-II was detected in 6.25% of the systemically healthy individuals, whereas no positive cases were reported among the systemically affected individuals. This contrast might contradict the conventional expectation that systemic conditions might predispose individuals to increased viral susceptibility, suggesting an altered environment that might be less hospitable to HHV colonization and persistence.

Despite viral presence, the subjects maintained a clinically healthy periodontium, possibly due to the immunomodulatory effects of latent *Herpesviridae* infection, which may enhance host resistance to bacterial colonization [112,113]. The dynamic relationship between viral and bacterial colonization may play a relevant role, as herpesviruses may suppress certain bacterial species, while conversely, specific bacteria can also create conditions that support viral persistence [11,12,14,65,114,115]. The higher negativity found in the systemically affected individuals may indicate a microbial composition lacking viral enablers or the influence of systemic medications on oral immunity [12,116,117].

The observed findings highlight the complexity of *Herpesviridae* interactions in the oral microbiome. The lower presence of viral loads in clinically healthy periodontium may indicate that mere viral detection alone may not equate to pathogenicity. Instead, viral activity is likely modulated by the periodontal microenvironment, shaped by the bacterial profile, host immune responses, and systemic health.

In conclusion, the systemically compromised pediatric subjects with periodontal disease exhibited distinct viral profiles compared to those with a healthy periodontium. In the children with periodontitis or gingivitis, EBV and CMV were frequently detected, suggesting that these viruses may play a role in disease progression and immune dysfunction. HSV was predominantly associated with gingivitis, indicating its potential involvement in the inflammatory processes during the early stages of the disease.

In contrast, the systemically compromised children with a healthy periodontium tended to exhibit a more diverse or variable viral composition, as observed in the comparison with the viral profile of the systemically healthy pediatric subjects. These findings highlight the intricate interactions between systemic health, viral colonization, and periodontal conditions, emphasizing the need for further research to clarify the role of viral infections in pediatric periodontal disease development.

### 4.5. Fungal Profile in Systemically Compromised Pediatric Subjects

#### 4.5.1. Fungi in Gingivitis and Periodontitis Pediatric Subjects

In this systematic review, fungi were investigated in three pediatric subjects, representing 4.35% of the study population. Among these, *Candida albicans* was detected in one subject (33.33% of the tested population) with Fanconi anemia and diabetes mellitus. Fungal culture revealed a low count of *Candida albicans*, comprising 0.3% of the total microbial culture. Conversely, fungi were absent in the remaining two subjects with periodontitis (66.67%), diagnosed with DOCK8 deficiency and Papillon–Lefèvre syndrome, respectively.

The detection of *Candida albicans* in the subject with Fanconi anemia and diabetes mellitus suggests that systemic conditions, particularly those involving immune dysregulation and metabolic alterations, may create an environment favorable to fungal colonization [26,118]. Diabetes mellitus, for instance, is known to promote fungal growth due to hyperglycemia, which enhances *Candida* adherence to epithelial cells and biofilm formation [25,118]. Similarly, Fanconi anemia, a genetic condition associated with impaired hematopoiesis and immune dysfunction, may reduce the host’s ability to control fungal proliferation [26,119,120].

An additional factor that may have contributed to fungal colonization in this subject could be the accumulation of advanced glycation end products (AGEs), a hallmark of chronic hyperglycemia in diabetes [121,122,123]. AGEs interact with their receptor, RAGE, triggering inflammatory responses such as the release of pro-inflammatory cytokines, oxidative stress, and impaired healing, which may promote fungal colonization by weakening immune defenses [26,124,125]. Furthermore, the periodontitis inflammatory environment may have exacerbated the microbial dysbiosis, potentially facilitating the transition of *Candida albicans* from a commensal to a pathogenic state.

In contrast, fungi were not detected in the two other pediatric subjects with periodontitis, despite the presence of systemic conditions involving immune dysregulation that could potentially foster their colonization.

Interestingly, *Aggregatibacter actinomycetemcomitans*, detected in one subject, may have inhibited fungal hyphal growth and the transition to a pathogenic state through quorum-sensing molecules like autoinducer-2 production [126]. In the other subject, *Porphyromonas gingivalis* was identified, which, through its robust proteolytic activity, may have inhibited fungal colonization [11,127].

Additionally, acute inflammatory responses in periodontal tissues may suppress fungal colonization [25]. Immunodeficient states like DOCK8 deficiency are associated with exaggerated or dysregulated inflammatory responses [57,128], which may target fungi that were not detected while failing to control bacterial pathogens, thereby contributing to bacterial dysbiosis and periodontitis progression.

#### 4.5.2. Fungi in Clinically Healthy Periodontium

Fungi were not investigated in the subjects with clinically healthy periodontium in the present systematic review. However, it can be hypothesized that fungal colonization may have been absent in these subjects due to the interplay of intrinsic and extrinsic factors that maintained a stable microbial environment unfavorable to fungal proliferation.

*Candida* species are opportunistic pathogens that require favorable environmental conditions, such as an altered immune response, to transition from commensals to pathogens. For instance, in individuals with Down syndrome, factors like reduced salivary flow, altered saliva composition, and impaired immune response favor the growth of fungal species, including *Candida parapsilosis* and *Candida dubliniensis* [51,76,129]. Similarly, studies on HIV-positive individuals have demonstrated that fungi can proliferate in subgingival biofilms under conditions of immune suppression [130,131,132].

The microbial composition of the periodontal biofilm in the clinically healthy periodontium subjects may also have contributed to the absence of fungi. Certain bacterial species, such as *Porphyromonas gingivalis* and *Aggregatibacter actinomycetemcomitans*, have been known to influence fungal behavior by producing quorum-sensing molecules that inhibit fungal hyphal growth [18,127,133,134]. It can be hypothesized that the lower density and simpler composition of biofilm in the clinically healthy periodontium subjects may have created a less conducive environment to fungal colonization since low-complexity and load biofilms are associated with a reduced prevalence of fungi species, such as *Candida albicans* [25,132,135]. Furthermore, transient shifts in the periodontal microenvironment due to acute inflammatory responses, even in subclinical forms, might have suppressed yeast colonization, limiting their growth potential.

This dynamic interplay between host immune responses, systemic factors, and microbial interactions may influence fungal colonization in pediatric subjects with clinically healthy periodontium.

Systemic factors such as altered immunity could promote fungal growth under certain conditions, but effective host responses coupled with microbial interactions within the biofilm may counteract these effects. The pathogenic transition of *Candida* likely requires synergistic activity with other biofilm components [25,136], which may have been absent in the present study.

Further studies are therefore needed to elucidate the mechanisms by which systemic conditions and biofilm ecology influence fungal behavior and to clarify the role of fungi in the onset and progression of periodontal disease.

In conclusion, fungi, particularly *Candida albicans*, were detected in periodontitis associated with Fanconi anemia and diabetes mellitus. These conditions, involving immune dysregulation and metabolic alterations, may create an environment conducive to fungal colonization. Fungi were not detected in two other pediatric subjects with periodontitis, despite underlying immune issues, possibly due to the inhibitory effects of bacterial species like *Aggregatibacter actinomycetemcomitans* and *Porphyromonas gingivalis*, which may suppress fungal growth.

Fungi were not investigated in the subjects with clinically healthy periodontium; however, a stable microbial environment could likely have prevented fungal colonization, maintaining a healthy periodontal microbiome.

### 4.6. Periodontal Status and Related Microbial Profile According to Systemic Diseases, Disorders, and Syndromes

The present systematic review confirmed the alternative hypothesis, showing relevant differences in salivary and/or subgingival microbial profiles between the systemically compromised subjects with gingivitis or periodontitis and those with a clinically healthy periodontium. Specifically, greater microbial diversity was found in gingivitis or periodontitis, with a more extensive representation of bacterial, viral, and fungal species.

In contrast, in the subjects with clinically healthy periodontium, the microbial profile was less diverse; however, this lower variability, although, in some aspects, less characterized, could reflect a balance of microbial species within the oral microbiome, potentially indicative of a state of microbiological homeostasis.

These findings comprehensively highlight the notable heterogeneity in systemic conditions observed in the pediatric subjects assessed.

The present systematic review uncovered a wide range of systemic compromisation among the examined pediatric subjects, including primary immunodeficiencies and immune response disorders, genetic and congenital syndromes with immunological involvement, endocrine dysfunctions impairing immune homeostasis, and secondary immunosuppression due to infectious or nutritional causes. These conditions may profoundly shape the microbiological landscape and predispose affected individuals to dysbiosis and periodontal disease through a disrupted host–microbe interplay [48,137]. The balance between immune surveillance and microbial colonization might appear particularly fragile in these patients, where impaired defense mechanisms may create an environment that favors the persistence of opportunistic pathogens.

#### 4.6.1. Periodontal Status and Related Microbial Profile in Dock8 Deficiency

Within this context, DOCK8 deficiency, characterized by a primary immunodeficiency marked by defective immune cell migration, impaired cytotoxic responses, and heightened susceptibility to persistent infections, may foster an environment where pathogenic microorganisms evade clearance, dysbiosis intensifies, and inflammatory pathways become dysregulated, driving both oral and systemic manifestations.

The present dataset identified *Porphyromonas gingivalis*, *Treponema denticola*, and *Tannerella forsythia*, suggesting a dysbiotic shift predisposing the host to periodontal disease. These anaerobic, Gram-negative bacteria can actively evade immune defenses through phagocytosis inhibition, proteolytic gingipains, and complement modulation [14,58,59,60]. Their persistence in a DOCK8-deficient pediatric subject may underscore the failure of T-cells, NK cells, and dendritic cells to mount an effective counter-response.

Beyond immune dysfunction, DOCK8 deficiency may compromise collagen homeostasis, potentially increasing periodontal vulnerability [138]. As DOCK8 regulates actin cytoskeleton dynamics and extracellular matrix integrity, its impairment functionality could compromise the structural integrity of collagen within the periodontal ligament, rendering it more susceptible to degradation [138,139]. This, combined with the proteolytic activity exerted by gingipains of *Porphyromonas gingivalis*, may accelerate connective tissue degradation and the loss of periodontal attachment and exacerbate disease progression [115,140].

Additionally, *Capnocytophaga species* (*C. gingivalis*, *C. sputigena*, *C. ochracea*) were detected, which, in neutrophil dysfunction, may exacerbate periodontal disease. The presence of *Campylobacter concisus*, a pathogen implicated in both gastrointestinal and oral inflammatory diseases [141], further raises the possibility that dysbiosis in DOCK8 deficiency may not be confined to the oral cavity but could extend to the gastrointestinal tract. This aligns with clinical observations of DOCK8-deficient patients frequently suffering from chronic gastrointestinal infections [138,139], supporting the hypothesis of an oral–gut axis of microbial dysregulation, where the characteristic immunodeficiency of these individuals may predispose them to oral dysbiosis and broader systemic microbial imbalances.

The presently found viral profile also reflected the precarious immune balance in the context of periodontitis.

The detection of HSV-1 reinforces the well-documented susceptibility of DOCK8-deficient individuals to persistent herpesvirus infections, likely due to impaired T-cell memory and cytotoxic responses.

Although EBV and CMV were not investigated, DOCK8 deficiency has been associated with EBV-driven lymphoproliferative disorders and CMV-related opportunistic infections, suggesting that these viruses may still be present at undetectable levels or may emerge in later stages of disease progression [138].

Although no fungal colonization was observed, DOCK8 deficiency may predispose individuals to recurrent mucocutaneous candidiasis due to TH17 and IL-17 signaling defects, essential in antifungal immunity [138,139]. The currently found absence of *Candida species* may not preclude its potential involvement in periodontitis, as fungal colonization in these patients may exhibit episodic or transient patterns influenced by host immunity, environmental factors, and the inflammatory profile at a given time.

The atopic phenotype in DOCK8 deficiency, through increased IgE levels and eosinophilic inflammation, may compromise epithelial barriers and facilitate microbial translocation [138,139], favoring, in turn, periodontitis development and/or progression. Moreover, eosinophilic inflammation and IL-4-driven responses impair cytotoxic immunity, selectively favoring viral persistence [139,142] and putatively explaining the link between atopy and *Herpesviridae*.

Although found in periodontitis, due to several immunological derangements, DOCK8 deficiency may also contribute to broader periodontal pathologies, including gingivitis and necrotizing gingivitis. The impaired immune surveillance, compounded by an atopic profile and potential collagen alterations [138,139], may facilitate an environment where pathogenic microorganisms proliferate, inciting aberrant inflammatory responses that may lead to diverse periodontal disease presentations.

#### 4.6.2. Periodontal Status and Related Microbial Profile in HIV-Positive Subjects

In the HIV-positive children with gingivitis, the viral profile was investigated despite the absence of data concerning bacterial and fungal species. Among *Herpesviridae*, CMV, HSV-I, and HSV-II were found, whereas EBV and VZV were not identified. The bacterial profile was not investigated in the present systematic review in the subjects with HIV; however, immunocompromised individuals are often associated with an altered microbiota, including red and orange complex bacteria species, which are known to drive inflammatory processes in periodontal disease [31,48,143]. The presence of these pathogens in HIV-positive individuals may be facilitated by immune dysregulation, particularly reduced neutrophil function and altered cytokine responses, leading to an environment that might favor microbial overgrowth and chronic inflammation [30].

The viral findings in the gingivitis pediatric subjects underscored the role of herpesviruses in periodontal disease progression, particularly in the context of HIV-related immunosuppression. Indeed, CMV may contribute to tissue destruction by inducing pro-inflammatory cytokine release, impairing fibroblast function, and facilitating bacterial colonization [79,92]. Similarly, HSV-I and HSV-II may be linked to episodic reactivations that may exacerbate periodontal inflammation.

Notably, herpesviruses, specifically CMV, were also found in the HIV-positive individuals with a clinically healthy periodontium, while HSV-I and EBV were absent. This suggests that viral persistence might be a stable component of the oral environment in immunocompromised patients, even without overt clinical inflammation. The presence of CMV in clinically healthy tissues may reflect its ability to establish latent infections within gingival fibroblasts and endothelial cells, potentially predisposing individuals to future periodontal breakdown when triggered by immune fluctuations or bacterial dysbiosis.

Although fungal profiling was not performed, *Candida* species are frequently detected in HIV-positive individuals [144]. The immunosuppressive effects of HIV might facilitate fungal colonization, further contributing to dysbiosis and increased susceptibility to opportunistic infections.

#### 4.6.3. Periodontal Status and Related Microbial Profile in Malnutrition/Kwashiorkor

The microbial profile observed in the pediatric patients with malnutrition/kwashiorkor was reported in necrotizing gingivitis. Although bacterial profiling was not directly assessed, necrotizing gingivitis is predominantly associated with *Spirochetes* and *Bacteroides* species [145]. Chronic malnutrition impairs the hypothalamic–pituitary–adrenal axis, leading to sustained hypercortisolism, which limits neutrophil recruitment and phagocytic function [146], potentially facilitating the persistence of pathogenic biofilms. Additionally, cortisol may serve as a metabolic substrate for *Bacteroides*, supporting their proliferation within the gingival niche [145,147].

Moreover, sarcopenia and vitamin deficiencies further exacerbate immune dysfunction [146], creating an environment conducive to microbial dysbiosis and periodontal inflammation.

The viral profile was characterized by the detection of CMV, EBV, HSV, and HHV-6, while HPV and Parvovirus B-19 were absent, suggesting that nutritional deprivation likely modulated host–pathogen interactions, potentially shaping the microbiological environment in necrotizing gingivitis.

Malnutrition-induced T-cell dysfunction and chronic systemic inflammation may favor *Herpesviridae* reactivation, therefore explaining their presence [146]. Moreover, the co-infections of several HHVs may indicate a complex interplay in which viral reactivation may be mutually reinforcing.

Fungal colonization, particularly by *Candida species*, was not investigated, but it may likely represent another dimension of microbial dynamics in malnutrition. Compromised epithelial barriers and reduced salivary IgA weaken host defenses, allowing increased fungal adherence and persistence [145]. Dysregulated iron homeostasis in malnutrition may further promote fungal overgrowth, exacerbating oral dysbiosis [148,149].

Interestingly, despite the potential for immune dysfunction to facilitate microbial persistence, this systematic review also identified subjects with malnutrition who exhibited a clinically healthy periodontium. Although only the viral profile was characterized in these individuals, no periodontal diseases were observed. This suggests that malnutrition may also exert selective pressures on the oral microbiome, potentially limiting colonization by key periodontal pathogens through nutrient competition or immune-mediated microbial interactions. The host immune response, while compromised, may still exert regulatory control over biofilm dynamics, preventing the microbial shifts necessary to trigger disease. Additionally, competition within the biofilm itself may influence the balance between commensals and pathogens, maintaining a state of microbial homeostasis despite systemic immunosuppression.

#### 4.6.4. Periodontal Status and Related Microbial Profile in Hydroa Vacciniforme

Hydroa vacciniforme, a rare lymphoproliferative disorder affecting children, was found to be associated with periodontitis. In the present study, only the viral profile was characterized, revealing the presence of EBV, HHV-6, and HHV-7 in a single individual. EBV leads to chronic inflammation and immune activation [21,74], potentially compromising periodontal homeostasis by altering cytokine responses and impairing local immune surveillance. HHV-6 and HHV-7, both lymphotropic viruses, may have contributed to immune dysregulation, potentially leading to periodontal disease progression. Bacterial and fungal profiles were not investigated; however, it might be likely that immune dysfunction in this subject may have created a favorable environment for pathogens, bacteria, and opportunistic fungi colonization, further contributing to oral microbiome imbalances and disease progression.

#### 4.6.5. Periodontal Status and Related Microbial Profile in Papillon–Lefèvre

Papillon–Lefèvre syndrome was investigated in a periodontitis subject included in this review. The viral profile revealed CMV and EBV-I, likely suggesting a viral dysbiosis that may exacerbate periodontal inflammation.

Interestingly, *Porphyromonas gingivalis* was not detected, potentially due to the altered immune landscape in Papillon–Lefèvre, where severe neutrophil dysfunction may create an environment that disrupts the ecological niche required for its persistence [115,140]. Conversely, the orange complex species *Prevotella intermedia*/*nigriscens* and *Fusobacterium nucleatum* were found, potentially taking advantage of the neutrophil dysfunction. The presence of *Aggregatibacter actinomycetemcomitans* may have exacerbated periodontitis progression due to leukotoxin production and other virulence factors impairing immune cells [19,20].

Fungal species were absent, which may suggest that the altered immune environment might not favor fungal colonization, creating a more conducive bacterial–viral synergy rather than a fungal persistence ecological niche.

#### 4.6.6. Periodontal Status and Related Microbial Profile in Down Syndrome

The characterization of microbial profiles in pediatric subjects with Down syndrome and periodontal disease was underexplored.

Although bacteria were not characterized in the present systematic review, periodontal pathogens such as *Porphyromonas*, *Treponema*, *Tannerella*, and *Aggregatibacter* have been identified in individuals with Down syndrome-associated periodontitis [150]. Additionally, emerging pathogenic taxa, like *Peptostreptococcus*, *Filifactor*, *Fretibacterium*, and *Desulfobulbus*, have also been associated with periodontitis in Down syndrome subjects [150].

The systemic compromise in Down syndrome may influence the composition of the oral microbiome and pathogenicity. The characteristic immunological impairments, such as altered neutrophil function and reduced salivary flow, could facilitate the colonization and proliferation of these periodontal pathogens [29,51,53]. Therefore, the dysbiotic transition may exacerbate inflammatory responses, leading to the early onset and rapid progression of periodontal disease frequently observed in this population.

In the present systematic review, the viral profile revealed the presence of CMV, EBV, and HSV, with one case of co-infection with CMV and HSV. The presence of these viruses may further complicate periodontal status in individuals with Down syndrome. Poor oral hygiene, congenital dental anomalies, and compromised immune responses create a conducive environment for viral persistence and reactivation, potentially amplifying periodontal tissue destruction [53,54,150,151].

Although fungal content was not reported in this study, it is plausible that the dynamic interactions within the oral environment, including fungal species, may contribute to periodontal disease pathogenesis. Individuals with Down syndrome often exhibit a higher prevalence of *Candida* species [150,152], which, in conjunction with bacterial pathogens, may modulate the host’s immune inflammatory response, leading to enhanced periodontal breakdown.

The microbiological profile of healthy periodontium pediatric subjects with Down syndrome was not currently investigated; however, Down syndrome individuals with clinically healthy periodontium may harbor pathogenic microbiota similar to those with periodontal disease [150,152]. This phenomenon could be attributed to inherent immunological deficiencies and unique oral environmental factors in Down syndrome, predisposing them to periodontal pathogens independent of clinical periodontal status.

#### 4.6.7. Periodontal Status and Related Microbial Profile in Fanconi Anemia and Diabetes Mellitus

The microbial profile that emerged in the currently investigated Fanconi anemia and diabetes mellitus pediatric subjects might explain potential implications for periodontal disease progression.

The bacterial analysis identified *Campylobacter* species, *Fusobacterium* species, *Parvimonas micra*, *Aggregatibacter actinomycetemcomitans*, *Fusobacterium*, and *Parvimonas micra*, suggesting a microbial environment favoring inflammation and immune evasion. Given that both Fanconi anemia and diabetes are characterized by systemic oxidative stress and immune dysfunction [37,120], the proliferation of these bacterial species may be facilitated by an impaired host response, reduced antimicrobial peptide activity, and an altered redox balance in oral tissues.

The viral profile revealed the presence of active CMV and HSV. Increased susceptibility to viral infections could be linked to an immunocompromised status, where defective T-cell responses and chronic inflammation may allow viral reactivation. Specifically, the compromised immune function and defective DNA repair mechanisms in Fanconi anemia and the related oxidative stress may contribute to a dysbiotic oral microenvironment, potentially favoring viral persistence and increasing susceptibility to secondary infections [27,121,125].

Fungal analysis detected the presence of *Candida albicans*. Hyperglycemia in diabetes mellitus provides an optimal environment for fungal proliferation, as glucose-rich conditions may enhance fungal adhesion and biofilm formation [122,123]. Immune impairment may also create conditions favoring fungal overgrowth, further contributing to an imbalanced oral microbiota and increased inflammatory burden, potentially leading to periodontal disease.

### 4.7. Strengths, Limitations, and Future Directions

Several factors should be considered when interpreting the results of this systematic review due to its inherent limitations.

The studies included in this systematic review are relatively outdated, with publication dates ranging from 1997 to 2015. As a result, the microbial detection techniques used in these studies may not reflect the current technological advancements. Therefore, the detection methods employed may have either overestimated or underestimated the prevalence of pathogens, potentially failing to identify all relevant microbial strains.

Moreover, the lack of standardized and homogeneous sampling methods across the studies could have affected the consistency and comparability of the results.

Geographical differences in the study populations also represent a limitation. Some studies focused on participants from regions where certain infectious diseases, such as genetic disorders or syndromes, are more prevalent. This geographical diversity could have influenced the microbiological findings, limiting the generalizability of the results to other regions or populations. Additionally, environmental, genetic, and socioeconomic factors unique to these regions may have impacted the oral microbial composition, making the results less representative of populations from other geographic areas.

Another limitation that should be addressed is the potential risk of bias within the studies included in this systematic review. Variability in study design, sample sizes, and population characteristics may have introduced biases, undermining the strength of individual findings and the overall interpretation of the results.

Moreover, missing data, inconsistent reporting of standardized periodontal parameters, and lack of availability of crevicular and radiographic data further limited the evaluation of the findings.

Additionally, the underexplored role of fungi in systemically compromised pediatric subjects with gingivitis and periodontitis represents a significant gap in the literature, underscoring the need for further research in this area.

Despite these limitations, the present systematic review provides valuable insights into the current understanding of oral microbiota in pediatric patients with systemic conditions. Future research should focus on innovative approaches to expand on the current findings. Advanced technologies, such as artificial intelligence and machine learning, hold significant promise for analyzing large-scale microbiological data. These approaches could identify subtle patterns and interactions within microbial communities that traditional methods may overlook, as well as facilitate the development of predictive models for disease progression and personalized treatment strategies.

Moreover, the application of advanced multi-omics approaches, such as integrating genomics, proteomics, and metabolomics, holds great potential in providing a more comprehensive understanding of the oral microbiome in systemically compromised pediatric patients. This integrative approach could uncover previously unexplored microbial interactions, the role of host genetics, and the impact of systemic diseases on the microbiota.

The development of more sensitive, non-invasive diagnostic tools, such as biosensors or salivary biomarkers, could revolutionize the detection of oral pathogens and provide a more accessible means of early diagnosis and personalized interventions.

Furthermore, advances in the use of nanotechnology for targeted drug delivery systems to modulate the oral microbiome could open new insight into therapeutic strategies, particularly for pediatric patients with systemic conditions.

By addressing these directions, future research can advance our understanding of the complex interplay between systemic conditions and the oral microbiome, ultimately contributing to improved clinical outcomes for pediatric patients.

## 5. Conclusions

The microbiological profile in pediatric subjects with systemic compromised and periodontal conditions showed differences from those with healthy periodontium, suggesting a key role of systemic conditions in modulating the oral microbiota and in the progression of periodontal disease.

In subjects with gingivitis and periodontitis, the higher presence of specific pathogenic bacteria and viruses of the *Herpesviridae* family and fungi than in subjects with healthy periodontium suggests that the compromised immune status may favor a microbial environment more prone to dysbiosis and inflammatory progression. In contrast, in subjects with clinically healthy periodontium, the lower microbial diversity and the stability of the microbiota may suggest an immune response that is more effective in containing oral biofilm imbalances.

These findings highlighted the importance of considering systemic conditions not only as predisposing factors for periodontal disease but also as elements that influence microbial composition and its interactions.

The recognition of interactions among viruses, bacteria, and fungi in pediatric oral health emphasizes the importance of targeted preventive and therapeutic strategies, as well as a multidisciplinary approach that considers both systemic and local management of periodontal conditions in vulnerable pediatric populations.

## Figures and Tables

**Table 1:** Search strategy process for each database used.

Search Strategy:		
Keywords:		
Search Strategy: #1 AND #2 AND #3	#1—“human herpes virus” OR HHV OR herpesviridae OR herpes OR cytomegalovirus OR CMV OR HCMV OR “Epstein–Barr virus” OR EBV OR “herpes simplex virus” OR HSV OR “simplex virus” OR “herpes virus 4” OR “herpes virus 6” OR “herpes virus 7” OR “herpes virus 8”	
	#2—periodontitis OR “juvenile periodontitis” OR “aggressive periodontitis” OR “localized aggressive periodontitis” OR “generalized aggressive periodontitis” OR “prepubertal periodontitis” OR “localized prepubertal periodontitis” OR “generalized prepubertal periodontitis” OR “molar-incisor pattern periodontitis” OR gingivitis	
	#3—children OR adolescent OR infant OR juvenile OR young	
Databases:		
Scopus	MEDLINE/PubMed	Cochrane Library

**Table 2:** Eligibility criteria (inclusion/exclusion criteria) sorted by studies, population, and intervention(s).

	Inclusion Criteria	Exclusion Criteria
Studies	In the English language. No date, sample size, or gender restrictions. Case reports, case series, cross-sectional, case–control, prospective, and retrospective studies.	Not in the English language. In vitro studies, preclinical animal studies, reviews, conference papers, book chapters, and oral communications.
Population	Pediatric subjects (≤18 years of age). Systemically compromised (e.g., diseases, syndromes, congenital, metabolic, or immunological disorders). Diagnosed with gingivitis and/or periodontitis (according to AAP/EFP classifications [1]).	Adult subjects (>18 years of age). Not systemically compromised (e.g., diseases, syndromes, congenital, metabolic, or immunological disorders). Not diagnosed with gingivitis and/or periodontitis or not according to the AAP/EFP classifications [1].
Intervention(s)	Microbiological analyses of subgingival and/or salivary samples assessing the presence of HHV (and other viruses), bacteria, and fungi in at least one gingivitis and/or periodontitis site and assessment of clinical, radiographic, and crevicular periodontal parameters.	No microbiological analyses of subgingival and/or salivary samples assessing the presence of HHV, bacteria, and fungi and clinical, radiographic, and crevicular parameters in pediatric subjects (≤18 years of age).

**Table 3:** Characteristics of the included studies: population, periodontal status, and microbiological analysis in systemically compromised pediatric subjects with a clinically healthy periodontium, gingivitis, or periodontitis.

Studies	Systemically Compromised Pediatric Subjects with Gingivitis/Periodontitis				Systemically Compromised Pediatric Subjects with a Clinically Healthy Periodontium			
	Population	Periodontal Status		Microbiological Analysis	Population	Periodontal Status		Microbiological Analysis
Betts K., 2015 *J Clin Virol.* Case-report [34] Include Intramural Research Program	Population: n. 1 Mean age: 17 yo n. 1 Gender ratio: 1 F Country/Ethnicity: Lebanon n. 1 Comorbidities: DOCK8 deficiency n. 1 Anamnesis for infectious disease: HSV-I n. 1 Therapy for infectious disease: Valacyclovir n. 1	Periodontal diagnosis: periodontitis n. 1		N. of sample: n. 2 Type of sample: saliva n. 1, subgingival n. 1 Sampling methods: MD Time after periodontal treatment: none n. 1 Microbiological identification technique: PCR n. 1, culture n. 1 Sampled site: MD Target: MD	NA	NA		NA
		Crevicular parameters	MD					
		Radiographic parameters	MD					
		Crevicular parameters	MD					
Contreras A., 1997 *Oral Microbiol Immunol* Comparative study [35] Moderate risk Nestle Foundation Switzerland, grant from US Public Health Service	Population: n. 22 Mean age: 7.9 ± 2.6 yo, range 3–14 yo Gender ratio: MD Country/Ethnicity: Nigeria n. 22 Comorbidities: malnutrition/marasmic–kwashiorkor n. 22 Anamnesis for infectious disease: MD Therapy for infectious disease: MD	Periodontal diagnosis: necrotizing gingivitis n. 22		N. of sample: n. 44 Type of sample: subgingival n. 44 Sampling methods: sterile paper points n. 44 Time after periodontal treatment: none n. 22 Microbiological identification technique: nested PCR n. 22 Sampled site: necrotizing gingivitis sites n. 22 Target: IE gene, EBNA1 gene, gag gene, nucleocapsid protein gene, L1 gene n. 22	Population: n. 40 Mean age: 7.1 ± 1.4 yo, range 3–14 yo n. 40 Gender ratio: MD Country/Ethnicity: Nigeria n. 40 Comorbidities: malnutrition n. 40 Anamnesis for infectious disease: MD Therapy for infectious disease: MD	Periodontal diagnosis: clinically healthy periodontium n. 40		N. of sample: n. 80 Type of sample: subgingival n. 80 Sampling methods: sterile paper points n. 80 Time after periodontal treatment: none n. 40 Microbiological identification technique: nested PCR n. 80 Sampled site: interproximal periodontal sulci n. 40 Target: IE gene, EBNA1 gene, gag gene, nucleocapsid protein gene, L1 gene n. 40
		Clinical parameters	MD			Clinical parameters	MD	
		Radiographic parameters	MD			Radiographic parameters	MD	
		Crevicular parameters	MD			Crevicular parameters	MD	
Hanookai D., 2000 *J Periodontol* Case–control study [36] Low risk No funding	Population: n. 6 Mean age: 16.84 yo, range 16–17 yo Gender ratio: MD Country/Ethnicity: MD Comorbidities: Down syndrome n. 6 Anamnesis for infectious disease: MD Therapy for infectious disease: MD	Periodontal diagnosis: periodontitis n. 6		N. of sample: n. 18 Type of sample: subgingival n. 18 Sampling methods: sterile paper points n. 18 Time after periodontal treatment: none n. 6 Microbiological identification technique: nested PCR n. 6 Sampled site: deepest periodontitis sites n. 6 Target: IE gene, EBNA2 n. 6	NA			NA
		Clinical parameters	PPD ≥ 5 mm n. 6					
			FMBS (Ainamo and Bay, mean %) 29.45 ± 19.3 n. 6					
			FMPS (O’Leary, mean %) 96.67 ± 6.83 n. 6					
			CI-S (Greene–Vermillion, mean %) 0.29 ± 0.16 n. 6					
		Radiographic parameters	Bone loss (%) 33.3% n. 2; 50% n. 3; 16.7% n. 1					
		Crevicular parameters	MD					
Nowzari H., 2001 *J Periodontol* Case report [37] Include No funding	Population: n. 1 Mean age: 11 yo Gender ratio: 1 M Country/ethnicity: Caucasian n. 1 Comorbidities: Fanconi anemia, diabetes mellitus n. 1 Anamnesis for infectious disease: recurrent herpes labialis n. 1 Therapy for infectious diseases: MD	Periodontal diagnosis: periodontitis n. 1		N. of sample: n. 3 Type of sample: subgingival n. 3 Sampling methods: sterile paper points n. 3 Time after periodontal treatment: none n. 1 Microbiological identification technique: nested PCR n. 1, culture n. 1 Sampled site: periodontitis sites n. 1 Target: MD	NA			NA
		Clinical parameters	MD					
		Radiographic parameters	MD					
		Crevicular parameters	MD					
Otero R.A., 2015 *Rev Inst Med Trop Sao Paulo* Case–control study [38] Low risk CNPq, CAPES, FAPERJ, Brazil	Population: n. 35 Mean age: N/D Gender ratio: N/D Country/ethnicity: MD Comorbidities: HIV n. 35 Anamnesis for infectious disease: HIV n. 35 Therapy for infectious diseases: MD	Periodontal diagnosis: gingivitis n. 35		N. of sample: n. 35 Type of sample: saliva n. 35 Sampling methods: sterile container n. 35 Time after periodontal treatment: none n. 35 Microbiological identification technique: PCR n. 35 Sampled site: MD Target: MD	Population: n. 13 Mean age: N/D Gender ratio: N/D Country/ethnicity: MD Comorbidities: HIV n. 13 Anamnesis for infectious disease: HIV n. 13 Therapy for infectious diseases: MD	Periodontal diagnosis: Clinically healthy periodontium n. 13		N. of sample: n. 13 Type of sample: saliva n. 13 Sampling methods: sterile container n. 13 Time after periodontal treatment: none n. 13 Microbiological identification technique: PCR n. 13 Sampled site: MD Target: MD
		Clinical parameters	BoP 1 n. 35			Clinical parameters	MD	
		Radiographic parameters	MD			Radiographic parameters	MD	
		Crevicular parameters	MD			Crevicular parameters	MD	
Satoh T., 2010 *Acta Derm Venereol* Case report [39] Include No funding	Population: n. 1 Mean age: 8 yo Gender ratio: 1 F Country/Ethnicity: MD Comorbidities: Hydroa Vacciniforme n. 1 Anamnesis for infectious disease: none n. 1 Therapy for infectious diseases: NA	Periodontal diagnosis: periodontitis n. 1		N. of sample: n. 2 Type of sample: subgingival n. 1, saliva n. 1 Sampling methods: MD Time after periodontal treatment: none n. 1 Microbiological analysis technique: nested PCR n. 1 Sampled site: periodontitis sites n. 1 Target: MD	NA	NA		NA
		Clinical parameters	MD					
		Radiographic parameters	MD					
		Crevicular parameters	MD					
Velazco C.H., 1999 *J Clin Periodontol.* Case report [40] Include No funding	Population: n. 1 Mean age: 11 yo Gender ratio: 1 F Country/Ethnicity: MD Comorbidities: Papillon–Lefèvre syndrome n. 1 Anamnesis for infectious disease: MD Therapy for infectious diseases: MD	Periodontal diagnosis: periodontitis n. 1		N. of sample: n. 3 Type of sample: subgingival n. 3 Sampling methods: sterile paper points n. 3 Time after periodontal treatment: none n. 3 Microbiological analysis technique: culture n. 1, nested PCR n. 1 Sampled site: deepest periodontal pockets n. 1 Target: 16S rRNA, IE gene EBNA2 n. 1	NA	NA		NA
		Clinical parameters	PPD 6–9 mm					
		Radiographic parameters	N/D					
		Crevicular parameters	MD					
Yildirim S., 2006 *Oral Microbiol Immunol.* Case series [41] Include No funding	Population: n. 2 Mean age: 7.5 yo, range 13–6 yo Gender ratio: 1 M/1 F Country/Ethnicity: MD Comorbidities: Kostmann syndrome n. 2 Anamnesis for infectious disease: MD Therapy for infectious diseases: MD	Periodontal diagnosis: periodontitis n. 2		N. of sample: n. 4 Type of sample: subgingival n. 2, saliva n. 2 Sampling methods: sterile toothbrush n. 2 Time after periodontal treatment: none n. 2 Microbiological analysis technique: PCR n. 2 Sampled site: MD Target: MD	NA	NA		NA
		Clinical parameters	MD					
		Radiographic parameters	MD					
		Crevicular parameters	MD					

**Abbreviations:** number “n.”, male “M”, female “F”, years old “yo”, missing data “MD”, not applicable “NA”, not defined “N/D, Immediate Early “IE”, Pocket Probing Depth “PPD”, Bleeding on Probing “BoP”, Full Mouth Bleeding Score “FMBS”, Full Mouth Plaque Score “FMPS”, Calculus Index-Simplified “CI-S”, Human Immunodeficiency Virus “HIV”, Dedicator Of Cytokinesis 8 “DOCK 8”.

**Table 4:** Salivary and/or subgingival microbial profile of systemically compromised pediatric subjects with a clinically healthy periodontium, gingivitis, or periodontitis.

Study	Microorganisms (Investigated)	Outcome(s)							
		Pediatric Subjects with Gingivitis/Periodontitis				Pediatric Subjects with a Clinically Healthy Periodontium			
		Culture		PCR		Culture		PCR	
		Quality	Quantity	Quality	Quantity	Quality	Quantity	Quality	Quantity
Betts K., 2015 [34] Periodontitis Intervention group: n. 1 Microbiological analysis technique: PCR n. 1, culture n. 1		**Viral Profile**							
	HHV:								
	HSV	NA	NA	Positive n. 1	MD	NA	NA	NA	NA
	EBV	NA	NA	Negative n. 1	MD	NA	NA	NA	NA
		**Bacterial Profile**							
	**Red complex species**								
	*Porphyromonas gingivalis*	Positive n. 1	MD	None	None	NA	NA	NA	NA
	*Treponema denticola*	Positive n. 1	MD	None	None	NA	NA	NA	NA
	**Orange complex species**								
	*Streptococcus constellatus*	Positive n. 1	MD	None	None	NA	NA	NA	NA
	**Yellow complex species**								
	*Streptococcus oralis*	Positive n. 1	MD	None	None	NA	NA	NA	NA
	**Green complex species**								
	*Capnocytophaga gingivalis*	Positive n. 1	MD	None	None	NA	NA	NA	NA
	*Capnocytophaga sputigena*	Positive n. 1	MD	None	None	NA	NA	NA	NA
	*Capnocytophaga ochracea*	Positive n. 1	MD	None	None	NA	NA	NA	NA
	*Campylobacter concisus*	Positive n. 1	MD	None	None	NA	NA	NA	NA
	**Blue complex species**								
	*Actinomyces georgiae*	Positive n. 1	MD	None	None	NA	NA	NA	NA
		**Fungal Profile**							
	*Fungi*	Negative n. 1	MD	None	None	NA	NA	NA	NA
Contreras A., 1997 [35] Necrotizing gingivitis Intervention group n. 22 Microbiological analysis technique: nested PCR n. 22 Control group: n. 40 Microbiological analysis technique: nested PCR n. 40		**Viral Profile**							
	HHV:								
	CMV	NA	NA	Positive n. 13 Negative n. 9	MD	NA	NA	Positive n. 1 Negative n. 39	MD
	EBV-I	NA	NA	Positive n. 6 Negative n. 16	MD	NA	NA	Positive n. 2 Negative n. 38	MD
	HSV	NA	NA	Positive n. 5 Negative n. 17	MD	NA	NA	Positive n. 1 Negative n. 39	MD
	HHV-6	NA	NA	Positive n. 1 Negative n. 21	MD	NA	NA	Negative n. 40	MD
	HHV Co-infections of positive samples:								
	N/D two HHV viruses	n. 6				None n. 40			
	N/D three HHV viruses	n. 2				None n. 40			
	Other viruses:								
	HPV	NA	NA	Negative n. 22	MD	NA	NA	Negative n. 40	MD
Hanookai D., 2000 [36] Periodontitis Intervention group: n. 6 Microbiological analysis technique: nested PCR n. 6		**Viral Profile**							
	HHV:								
	CMV	NA	NA	Positive n. 2 Negative n. 4	MD	NA	NA	NA	NA
	EBV-I	NA	NA	Positive n. 2 Negative n. 4	MD	NA	NA	NA	NA
	HSV	NA	NA	Positive n. 1 Negative n. 5	MD	NA	NA	NA	NA
	HHV Co-infections of positive samples:								
	CMV + HSV	n. 1							
Nowzari H., 2001 [37] Periodontitis Intervention group n. 1 Microbiological analysis technique: culture n. 1 nested PCR n. 1		**Viral Profile**							
	HHV:								
	CMV	NA	NA	Positive n. 1	MD	NA	NA	NA	NA
	CMV DNA (productive/active infection)	NA	NA	Positive n. 1	MD	NA	NA	NA	NA
	CMV m-RNA (latent infection)	NA	NA	Negative n. 1	MD	NA	NA	NA	NA
	HSV	NA	NA	Positive n. 1	MD	NA	NA	NA	NA
		**Bacterial Profile**							
	**Orange complex species**								
	*Campylobacter species*	Positive n. 1	2.2% * n. 1	None	None	NA	NA	NA	NA
	*Fusobacterium species*	Positive n. 1	7.9% * n. 1	None	None	NA	NA	NA	NA
	*Parvimonas micra*	Positive n. 1	3.4% * n. 1	None	None	NA	NA	NA	NA
	**Outlier bacterial species**								
	*Aggregatibacter actinomycetemcomitans*	Positive n. 1	1.1% * n. 1	None	None	NA	NA	NA	NA
		**Fungal Profile**							
	*Candida albicans*	Positive n. 1	0.3% * n. 1	None	None	NA	NA	NA	NA
Otero R.A., 2015 [38] Gingivitis n. 35 Intervention group: n. 35 Microbiological analysis technique: PCR n. 35 Control group n. 13 Microbiological analysis technique: PCR n. 13		**Viral Profile**							
	HHV:								
	HSV-I	NA	NA	Positive n. 3 Negative n. 32	MD	NA	NA	Negative n. 13	
	HSV-2	NA	NA	Positive n. 2 Negative n. 33	MD	NA	NA	Negative n. 13	
	CMV	NA	NA	Positive n. 5 Negative n. 30	MD	NA	NA	Positive n. 7 Negative n. 6	
	EBV	NA	NA	None n. 35	MD	NA	NA	Negative n. 13	None
	VZV	NA	NA	None n. 35	MD	NA	NA	Negative n. 13	None
	Other viruses:								
Satoh T., 2010 Nishizawa A., 2010 [39] Periodontitis Intervention group: n. 1 Microbiological analysis technique: nested PCR n. 1		**Viral Profile**							
	HHV:								
	EBV	NA	NA	Positive n. 1	NA	NA	NA	NA	NA
	HHV-6	NA	NA	Positive n. 1	NA	NA	NA	NA	NA
	HHV-7	NA	NA	Positive n. 1	NA	NA	NA	NA	NA
	Other viruses:								
	HPVB-19	NA	NA	Positive n. 1	NA	NA	NA	NA	NA
Velazco C.H., 1999 [40] Periodontitis Intervention group: n. 1 Microbiological analysis technique: culture n. 1 nested PCR n. 1		**Viral Profile**							
	HHV:								
	CMV	NA	NA	Positive n. 1	MD	NA	NA	NA	NA
	EBV-I	NA	NA	Positive n. 1	MD	NA	NA	NA	NA
		**Bacterial Profile**							
	**Red complex species**								
	*Porphyromonas gingivalis*	Negative n. 1	0.00% * n. 1	Positive n. 1	MD	NA	NA	NA	NA
	*Tannerella forsythia*	Negative n. 1	0.00% * n. 1	Negative n. 1	MD	NA	NA	NA	NA
	**Orange complex species**								
	*Prevotella intermedia*/*nigriscens*	Positive n. 1	16.40% * n. 1	Positive n. 1	MD	NA	NA	NA	NA
	*Parivimonas micra*	Positive n. 1	10.06% * n. 1	MD	MD	NA	NA	NA	NA
	*Eubacterium species*	Negative n. 1	0.00% * n. 1	None	None	NA	NA	NA	NA
	*Fusobacterium nucleatum*	Positive n. 1	14.30% * n. 1	MD	MD	NA	NA	NA	NA
	*Campylobacter species*	Negative n. 1	0.00% * n. 1	Negative n. 1	MD	NA	NA	NA	NA
	**Green complex species**								
	*Eikenella corrodens*	Positive n. 1	0.80% * n. 1	Positive n. 1	MD	NA	NA	NA	NA
	**Outlier bacteria species**								
	*Aggregatibacter actinomycetemcomitans*	Positive n. 1	3.40% * n. 1	Positive n. 1	MD	NA	NA	NA	NA
	*Enteric Gram-negative rods*	Negative n. 1	0.00% * n. 1	None	None	NA	NA	NA	NA
	*β-hemolytic streptococci*	Negative n. 1	0.00% * n. 1	None	None	NA	NA	NA	NA
		**Fungal Profile**							
	*Fungi*	Negative n. 1	0.00% * n. 1	None	None	NA	NA	NA	NA
Yildirim M., 2006 [41] Periodontitis Intervention group: n. 2 Microbiological analysis technique: PCR n. 2		**Viral Profile**							
	HHV:								
	EBV	NA	NA	Positive n. 2	MD	NA	NA	NA	NA
	CMV	NA	NA	Negative n. 2	MD	NA	NA	NA	NA
		**Bacterial Profile**							
	Red complex species								
	*Porphyromonas gingivalis*	None	None	Negative n. 2	MD	NA	NA	NA	NA
	*Tannerella forsythia*	None	None	Negative n. 2	MD	NA	NA	NA	NA
	**Orange complex species**								
	*Prevotella intermedia*	None	None	Negative n. 2	MD	NA	NA	NA	NA
	*Campylobacter rectus*	None	None	Positive n. 2	MD	NA	NA	NA	NA
	**Outlier species**								
	*Aggregatibacyer actinomycetemcomitans*	None	None	Negative n. 2	MD	NA	NA	NA	NA

Abbreviation: not applicable “NA”, missing data “MD”, human herpesvirus HHV, CytoMegaloVirus “CMV”, Polymerase Chain Reaction “PCR”, Epstein–Barr virus “EBV”, Epstein–Barr type I “EBV-I”, percentage “%” of total counts “*”, herpes simplex virus “HSV”, human herpesvirus 6 “HHV-6”, Human Papilloma Virus “HPV”, Varicella-Zoster virus “VZV”, Human Parvo Virus B-19 “ HPVB-19”.

## Data Availability

Data are available in the MEDLINE/PubMed, Scopus, Web of Science, and Cochrane Library.

## Supplementary Materials

The following supporting information can be downloaded at https://www.mdpi.com/article/10.3390/children12030375/s1: Supplementary File S1: Quality assessment. Table S1: Quality assessment of included nonrandomized studies according to ROBINS-1. First author, year, reference, ROBINS-1 bias domain, and quality assessment. Table S2: Quality assessment of included case reports according to JBI for case reports. First author, year, reference, JBI domain, and overall appraisal. Table S3: Quality assessment of included case series according to JBI for case series. First author, year, reference, JBI domain, and overall appraisal.
