# Supplementary material for: Periodontal Status and Herpesiviridae, Bacteria, and Fungi in Gingivitis and Periodontitis of Systemically Compromised Pediatric Subjects: A Systematic Review
Source: Children (Basel). 2025 Mar 17;12(3):375. doi: 10.3390/children12030375 (PMC11941093; doi:10.3390/children12030375)
Supplement: Supplementary file 1 [file children-12-00375-s001.zip › children-3510441-supplementary.pdf]

# Periodontal Status and Herpesiviridae, Bacteria, and Fungi in Gingivitis and Periodontitis of Systemically Compromised Pediatric Subjects: A Systematic Review

**Federica Di Spirito *, Massimo Pisano †, Maria Pia Di Palo †, Giuseppina De Benedetto, Iman Rizki, Gianluigi Franci *,‡ and Massimo Amato ‡**

Department of Medicine, Surgery and Dentistry, University of Salerno, Via S. Allende, 84081 Baronissi, Italy; pisano.studio@virgilio.it (M.P.); mariapia140497@gmail.com (M.P.D.P.); giusydb15@gmail.com (G.D.B.); i.rizki@studenti.unisa.it (I.R.); mamato@unisa.it (M.A.)
* Correspondence: fdispirito@unisa.it (F.D.S.); gfranci@unisa.it (G.F.)
† These authors contributed equally to this work.
‡ These authors contributed equally to this work.

**Supplementary File S1: Quality assessment**

Quality assessment of included nonrandomized studies of interventions (Table S1), using the Risk of Bias in Nonrandomized Studies of Interventions (ROBINS-1), of case reports (Table S2), using the Johanna Briggs Institute (JBI) for case reports, and of case series (Table S3) using the Johanna Briggs Institute (JBI) for case series.

**Table S1**. Quality assessment of included nonrandomized studies according to ROBINS-1. First Author, year, reference, ROBINS-1 bias domain, and quality assessment.

| | Conteras A., 1997 [35] | Hanookai D., 2000 [36] | Otero R.A, 2015 [38] |
|---|---|---|---|
| **Domain 1** | | | |
| 1.1 | N | N | N |
| 1.2 | NA | NA | NA |
| 1.3 | NA | NA | NA |
| 1.4 | NA | NA | NA |
| 1.5 | NA | NA | NA |
| 1.6 | NA | NA | NA |
| 1.7 | NA | NA | NA |
| 1.8 | NA | NA | NA |
| Domain 1 Judgment | Low risk | Low risk | Low risk |
| **Domain 2** | | | |
| 2.1 | N | N | N |
| 2.2 | NA | NA | NA |
| 2.3 | NA | NA | NA |
| 2.4 | NI | Y | Y |
| 2.5 | NA | NA | NA |
| Domain 2 Judgment | Moderate risk | Low risk | Low risk |
| **Domain 3** | | | |
| 3.1 | Y | Y | Y |
| 3.2 | PY | Y | Y |
| 3.3 | N | N | N |
| Domain 3 Judgment | Low risk | Low risk | Low risk |
| **Domain 4** | | | |

| | | | |
|---|---|---|---|
| 4.1 | N | N | N |
| 4.2 | NA | NA | NA |
| 4.3 | NA | NA | NA |
| 4.4 | NA | NA | NA |
| 4.5 | NA | NA | NA |
| 4.6 | NA | NA | NA |
| Domain 4 Judgment | Low risk | Low risk | Low risk |
| **Domain 5** | | | |
| 5.1 | Y | Y | Y |
| 5.2 | N | N | N |
| 5.3 | N | N | N |
| 5.4 | NA | NA | NA |
| 5.5 | NA | NA | NA |
| Domain 5 Judgment | Low risk | Low risk | Low risk |
| **Domain 6** | | | |
| 6.1 | N | N | N |
| 6.2 | Y | Y | Y |
| 6.3 | Y | Y | Y |
| 6.4 | N | N | N |
| Domain 6 Judgment | Low risk | Low risk | Low risk |
| **Domain 7** | | | |
| 7.1 | N | PN | N |
| 7.2 | PY | PN | N |
| 7.3 | N | N | N |
| Domain 7 Judgment | Moderate risk | Low risk | Low risk |
| **Quality assessment** | Moderate risk of bias | Low risk of bias | Low risk |

**Abbreviations:** yes “Y”; no “N”; Not Applicable “NA”; Probably yes “PY”; Probably no “PN”; No Information “NI”.

**Table S2**. Quality assessment of included case reports according to JBI for case reports. First Author, year, reference, JBI domain, and overall appraisal.

| | Case reports | | | | |
|---|---|---|---|---|---|
| | Betts K., 2015 [34] | Nowzari H., 2001 [37] | Velazco C.H., 1999 [40] | Yildirim S., 2006 [41] | Satoh T., 2010 [39] |
| 1 | Y | Y | Y | Y | Y |
| 2 | Y | Y | Y | Y | Y |
| 3 | N | Y | N | Y | Y |
| 4 | Y | Y | Y | Y | Y |
| 5 | Y | Y | Y | Y | NA |
| 6 | N | N | NA | Y | NA |
| 7 | N | N | N | Y | N |
| 8 | Y | Y | Y | Y | Y |
| Overall appraisal | Include | Include | Include | Include | Include |

**Abbreviations:** Yes, “Y”; No, “N”; Not Applicable, “NA”.

**Table S3.** Quality assessment of included case series according to JBI for case series. First Author, year, reference, JBI domain, and overall appraisal.

| Case series | |
|---|---|
| | Yildirim S., 2006 [41] |
| 1 | Y |
| 2 | Y |
| 3 | Y |
| 4 | NA |
| 5 | Y |
| 6 | Y |
| 7 | Y |
| 8 | Y |
| 9 | Y |
| 10 | NA |
| Overall appraisal | Include |

**Abbreviations:** Yes, "Y"; No, "N"; Not Applicable, "NA".
